# Supplementary material for: A cell state-specific metabolic vulnerability to GPX4-dependent ferroptosis in glioblastoma
Source: EMBO J. 2024 Aug 27;43(20):4492–521. doi: 10.1038/s44318-024-00176-4 (PMC11480389; doi:10.1038/s44318-024-00176-4)
Supplement: Supplementary file 1 — Table EV1 [file 44318_2024_176_MOESM1_ESM.pdf]

Table EV1: Common Differentially Expressed Genes in Persister Cells and N1IC Glioma Cells

N1IC_up ((l2fc≥1)	N1IC_down (l2fc≤-1)	N1IC_up (l2fc≥0.5)	N1IC_down (l2fc≤-0.5)	N1IC_up (l2fc>0)	N1IC_down (l2fc<0)
Acss1	Abhd2	Acss1	Abhd2	Acss1	Abhd2
Ankrd46	Cdk14	Ankrd46	Ago2	Ankrd46	Acaca
Arhgef28	Cdk17	App	Ank2	App	Ago2
Cacna1e	Cited2	Arhgef28	Apba2	Arhgef28	Ank2
Cald1	Cyfip1	Bcan	Ccdc92	Bcan	Apba2
Capg	Dennd2a	Bhlhe40	Cdk13	Bhlhe40	B3gat1
Chrm3	Evl	Cacna1e	Cdk14	Cacna1e	Cab39
Csrp1	Fbrsl1	Cald1	Cdk17	Cald1	Ccdc92
Ehd3	Fry	Capg	Cited2	Calm1	Cdk13
Fabp7	Fscn1	Ccdc174	Cnot10	Capg	Cdk14
Fam102a	Fzd1	Cdc5l	Col11a1	Ccdc174	Cdk17
Fam210b	Gab2	Chrm3	Cyfip1	Ccnd2	Cited2
Fam46a	Gpc1	Csrp1	Ddx39b	Cd47	Cldn11
Has2	Hmox1	Dct	Dennd2a	Cdc5l	Cnot10
Hes1	Hnrnpa0	Ehd3	Dpysl3	Chrm3	Col11a1
Hes5	Jag1	Emc2	Evl	Csrp1	Cyfip1
Hey1	Kansl1	Fabp7	Fam76b	Ctnnb1	Ddx39b
Hmcn1	Klf13	Fam102a	Fbrsl1	Dct	Dennd2a
Id3	Mib1	Fam210b	Fchsd2	Ehd3	Dpysl3
Ifit3	Mn1	Fam46a	Fry	Emc2	Epb41l2
Kbtbd11	Myt1	Has2	Fscn1	Fabp7	Evl
Litaf	Nav3	Hes1	Fzd1	Fam102a	Fam76b
Ncald	Ncor2	Hes5	Gab2	Fam210b	Fbrsl1
Pex26	Nova1	Hey1	Gatad1	Fam46a	Fchsd2
Pla2g4a	Ntn1	Hmcn1	Glud1	Frg1	Fry
Plp1	Pde3b	Id3	Gpc1	Gng12	Fscn1
Rassf4	Pdgfa	Ifit3	Hmox1	Has2	Fzd1
Runx3	Phyhipl	Kbtbd11	Hnrnpa0	Hes1	Gab2
Sat1	Ranbp9	Kcnj10	Jag1	Hes5	Gatad1
Serpine2	Sipa1l1	Lef1	Kansl1	Hey1	Glud1
Shc4	Sipa1l3	Litaf	Khdrbs3	Hmcn1	Gpc1
Sorl1	Tbkbp1	Mcam	Klf13	Id3	Gsap
Spats2l	Tnr	Ncald	Kpnb1	Ifit3	Hmox1
Tmc6	Ubfd1	Ndufa4	Magi2	Igsf3	Hnrnpa0
Trim2	Wdr38	Nes	Mbtd1	Kank1	Igfbp2
Zfhx4	Xylt1	Pex26	Mib1	Kbtbd11	Jag1
Zfp36l1	Ywhah	Pla2g4a	Mn1	Kcnj10	Kansl1
	Zbtb18	Plp1	Mtss1l	Lef1	Khdrbs3
	Zc3h4	Polr2b	Myt1	Litaf	Klf13
	Zcchc8	Ptn	Nav3	Lmo4	Kpnb1
	Zfand5	Rassf4	Nckap1	Mcam	Magi2
		Rsu1	Ncor2	Ncald	Mbtd1
		Runx3	Nova1	Ndufa4	Mib1
		Sat1	Ntn1	Nes	Mn1
		Serpine2	Olig1	Pdgfra	Msl2
		Shc4	Osbpl6	Pex26	Mtss1l
		Sorl1	Oxr1	Pla2g4a	Myt1
		Spats2l	Pde3b	Plp1	Nav3
		Tmc6	Pdgfa	Polr2b	Nckap1
		Trim2	Phyhipl	Psap	Ncor2
		Trio	Polg	Ptn	Nova1
		Ttyh1	Ppm1f	Rassf4	Ntn1
		Wbp4	Ptbp2	Rsu1	Olig1

		Zfhx4	Pten	Runx3	Osbpl6
		Zfp36l1	Ranbp9	Sat1	Oxr1
			Rhob	Serpine2	Pde3b
			Sema3d	Shc4	Pdgfa
			Sipa1l1	Slu7	Phyhipl
			Sipa1l3	Sorl1	Polg
			Sox6	Spats2l	Ppm1f
			Tbkbp1	Tbca	Ptbp2
			Tet2	Tcf4	Pten
			Tnr	Tmc6	Ranbp9
			Ubfd1	Trim2	Rhob
			Ubl3	Trio	Sema3d
			Wdr38	Ttyh1	Sipa1l1
			Xylt1	Ugp2	Sipa1l3
			Ywhah	Vmp1	Sox2
			Zbtb18	Wbp4	Sox6
			Zc3h4	Zc3h15	Tbkbp1
			Zcchc8	Zfhx4	Tet2
			Zfand5	Zfp36l1	Tnr
					Ubfd1
					Ubl3
					Wdr38
					Xylt1
					Ywhah
					Zbtb18
					Zc3h4
					Zcchc8
					Zfand5
