# Supplementary material for: A cell state-specific metabolic vulnerability to GPX4-dependent ferroptosis in glioblastoma
Source: EMBO J. 2024 Aug 27;43(20):4492–521. doi: 10.1038/s44318-024-00176-4 (PMC11480389; doi:10.1038/s44318-024-00176-4)
Supplement: Supplementary file 2 — Table EV2 [file 44318_2024_176_MOESM2_ESM.pdf]

Table EV2: Clinical and pathologic patient characteristics of organotypic slice culture specimens

Tissue ID	Age range	Sex	Location	Diagnosis	IDH1 Status	EGFR status	MGMT methylation
6181	30-39	F	Frontotemporal	AA	Mutant	Unamplified	Methylated
6328	70-79	F	Left frontal	GBM	Wildtype	Amplified	Methylated
6458	50-59	M	Right frontal	GBM	Wildtype	Amplified	Methylated
6528	60-69	F	Left temporal	GBM	Wildtype	Unamplified	Unmethylated
6534	50-59	F	Right temporal	GBM	Wildtype	Unamplified	Unmethylated
6545	30-39	M	Right Frontal	GBM	Wildtype	Unamplified	Unmethylated
