# Supplementary material for: A cell state-specific metabolic vulnerability to GPX4-dependent ferroptosis in glioblastoma
Source: EMBO J. 2024 Aug 27;43(20):4492–521. doi: 10.1038/s44318-024-00176-4 (PMC11480389; doi:10.1038/s44318-024-00176-4)
Supplement: Supplementary file 3 — Appendix [file 44318_2024_176_MOESM3_ESM.pdf]

TABLE OF CONTENTS

Appendix Figure S1-page 2-3
Appendix Figure S2-page 4-5
Appendix Figure S3-page 6-7
Appendix Figure S4-pages 8-9
Appendix Figure S5-pages 10-11
Appendix Figure S6-pages 12-13
Appendix Figure S7-pages 14-15
Appendix Figure S8-pages 16-17
Appendix Figure S9-pages 18-19
Appendix Figure S10-pages 20-21
Appendix Figure S11-pages 22-23
References-page 24

Appendix Figure S1 (associated with Figure 1): Radiographic, histopathologic, and transcriptomic features of the N1IC and p53 mouse glioma models

a. MRI demonstrates significantly slower tumor progression and increased latency in tumor formation in p53-/- N1IC mice with diffusely infiltrative tumors at 30 dpi and large necrotic and hemorrhagic tumors at 60 dpi. p53 tumors reach end stage around 30 dpi.
b. Representative H&E staining of endstage tumors demonstrate high grade histological features, including pseudopalisading necrosis and neo-angiogenesis in both models. Scale bars, 2 mm for panoramic view (top) and 100 µm for detailed inset (bottom).
c. Violin plot of AUCell persister scores in the two models, P-value via Welch's t test. Persister scores based on gene set described by Liau et al (Liau *et al*, 2017).
d. Heatmap of differentially expressed genes that are direct N1ICD targets in persister cells.

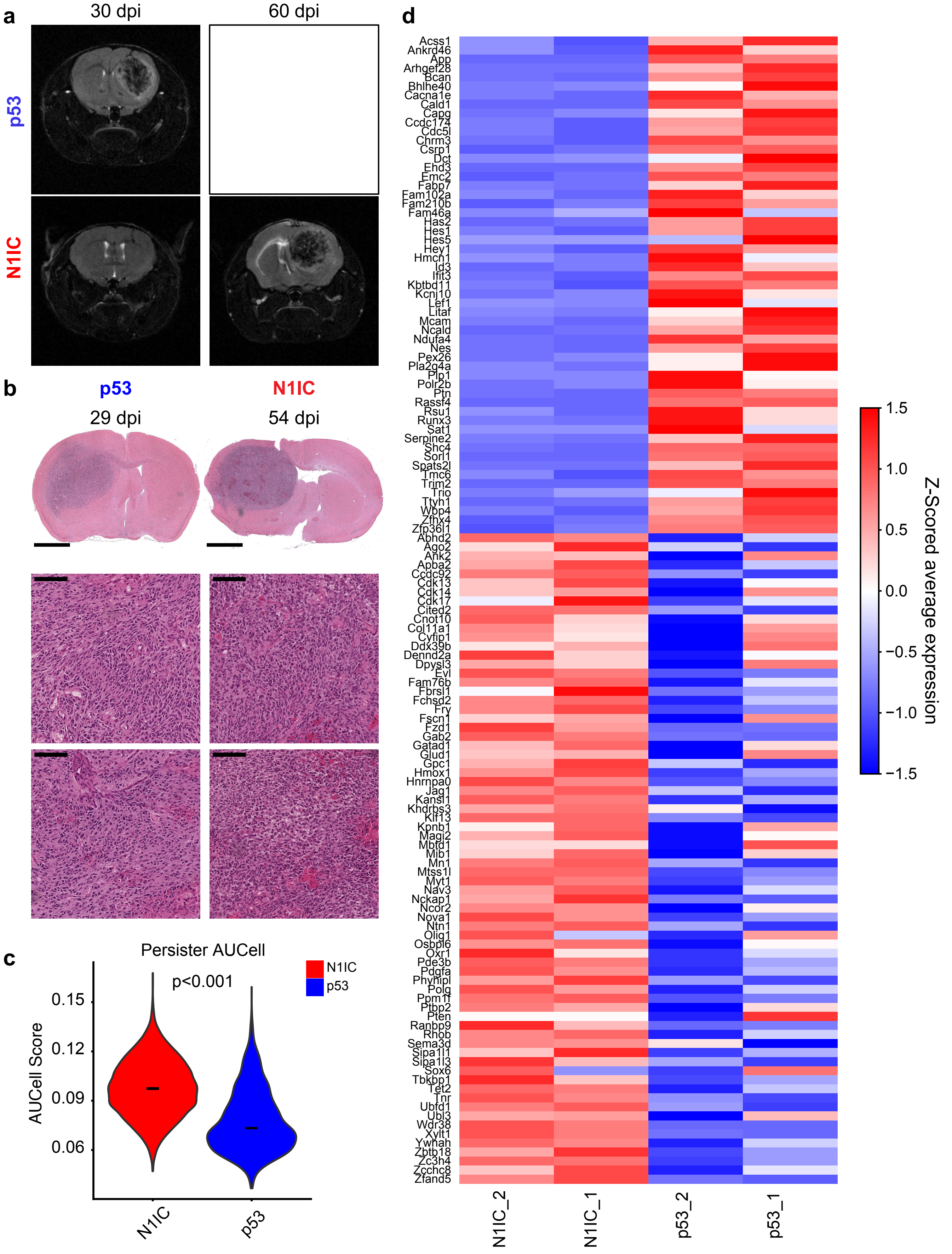
a
30 dpi
60 dpi
p53
N1IC
b
p53
N1IC
29 dpi
54 dpi
c
Persister AUCell
N1IC
p53
p<0.001
AUCell Score
0.15
0.12
0.09
0.06
N1IC
p53
d
Acss1
Ankrd46
App
Arhgef28
Bcan
Bhlhe40
Cacna1e
Cald1
Capg
Ccdc174
Cdc5l
Chrm3
Csrp1
Dct
Ehd3
Emc2
Fabp7
Fam102a
Fam210b
Fam46a
Has2
Hes1
Hes5
Hey1
Hmcn1
Id3
Ifit3
Kbtbd11
Kcnj10
Lef1
Litaf
Mcam
Ncald
Ndufa4
Nes
Pex26
Pla2g4a
Plp1
Polr2b
Ptn
Rassf4
Rsu1
Runx3
Sat1
Serpine2
Shc4
Sorl1
Spats2l
Tmc6
Trim2
Trio
Ttyh1
Wbp4
Zfhx4
Zfp36l1
Abhd2
Ago2
Ank2
Apba2
Ccdc92
Cdk13
Cdk14
Cdk17
Cited2
Cnot10
Col11a1
Cyfip1
Ddx39b
Dennd2a
Dpysl3
Evl
Fam76b
Fbrsl1
Fchsd2
Fry
Fscn1
Fzd1
Gab2
Gatad1
Glud1
Gpc1
Hmox1
Hnrnpa0
Jag1
Kansl1
Khdrbs3
Klf13
Kpnb1
Magi2
Mbtd1
Mib1
Mn1
Mtss1l
Myt1
Nav3
Nckap1
Ncor2
Nova1
Ntn1
Olig1
Osbpl6
Oxr1
Pde3b
Pdgfa
Phyhipl
Polg
Ppm1f
Ptbp2
Pten
Ranbp9
Rhob
Sema3d
Sipa1l1
Sipa1l3
Sox6
Tbkbp1
Tet2
Tnr
Ubfd1
Ubl3
Wdr38
Xylt1
Ywhah
Zbtb18
Zc3h4
Zcchc8
Zfand5
N1IC_2
N1IC_1
p53_2
p53_1
Z-Scored average expression
1.5
1.0
0.5
0.0
−0.5
−1.0
−1.5

Appendix Figure S2 (Associated with Figure 2): Differential activation of Notch signaling in primary cell cultures isolated from N1IC vs. p53 tumors

a. Genomic DNA PCR isolated from 6 different murine cell lines demonstrating persistence of the N1IC transgene in all three N1IC cell lines.
b. qRT-PCR of direct Notch downstream targets *Hey1*, *Hes1* and *Hes5* demonstrating upregulation of *Hey1/Hes5* in the N1IC cell line. Data obtained on a pair of N1IC/p53 cells pooled from n=3 independent experiments. P-values calculated via unpaired t test.
c. Immunofluorescence of N1IC and the tumor marker HA in a pair of p53/N1IC cells demonstrating nuclear localization of the intracellular portion of the Notch1 receptor in the N1IC cell line. All cells cultured *in vitro* expressed the HA marker. Scale bar, 10 μm.
d. Quantification of N1IC mean fluorescence intensity in p53 and N1IC cell lines, n=9 different fields per cell line. P-value via unpaired t test.

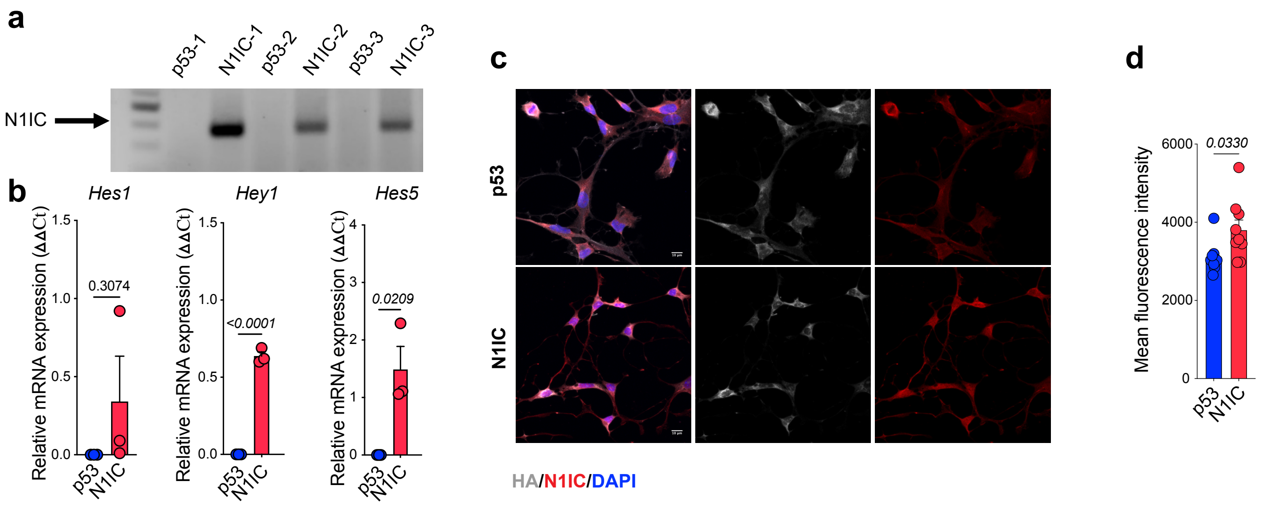
a
p53-1
N1IC-1
p53-2
N1IC-2
p53-3
N1IC-3
N1IC
b
Hes1
Hey1
Hes5
Relative mRNA expression (ΔΔCt)
0.3074
<0.0001
0.0209
p53
N1IC
c
p53
N1IC
HA/N1IC/DAPI
d
Mean fluorescence intensity
0.0330
p53
N1IC

Appendix Figure S3 (Associated with Figure 2): Metabolomic/lipidomic pathway analysis reveals differences between N1IC and p53 models

a. Metabolite Set Enrichment Analysis (MSEA) depicting significant ontologies from untargeted metabolomic LC-MS in p53 (blue) and N1IC (red) cell lines.
b. Combined transcriptomic/metabolomic pathway analysis in the N1IC model demonstrating activation of taurine/hypotaurine, pyruvate and pentose phosphate pathways. Select significant pathways are highlighted in red. Punctate line indicates cutoff level of significance ($p<0.05$).
c. KEGG-based diagram depicting compensatory upregulated genes encoding enzymes and enriched transcriptional metabolic pathways in the N1IC model. Significant genes/pathways in N1IC compared to p53 tumors highlighted in red. The AC-like model demonstrates ineffective cysteine/methionine metabolism secondary to high ROS production and decreased mitochondrial metabolic function, including decreased FAO, TCA cycle activity and ETC function based on functional/metabolomic analysis. FAO – fatty acid oxidation, TCA – tricarboxylic acid.
d. PCA of lipidomics data shows that the lipid profile segregates by model, in both the negative and positive mode.
e. Lipid ontology analysis demonstrating upregulation in TAG in the N1IC model with enrichment in lipids stabilizing the membrane in p53 cells. Blue – enriched in p53 cell cultures, red – enriched in N1IC cell cultures.

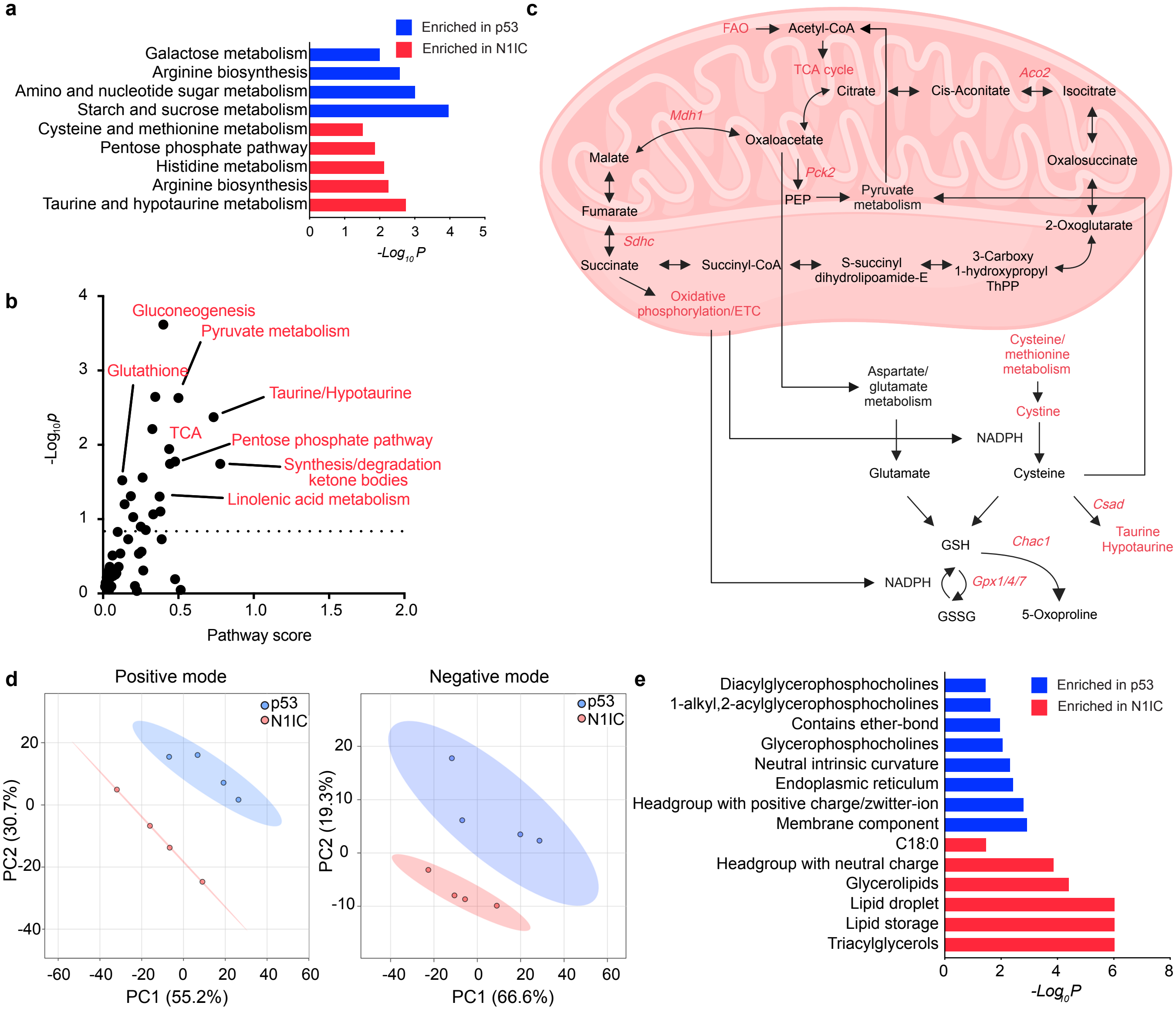
a
Enriched in p53
Enriched in N1IC
Galactose metabolism
Arginine biosynthesis
Amino and nucleotide sugar metabolism
Starch and sucrose metabolism
Cysteine and methionine metabolism
Pentose phosphate pathway
Histidine metabolism
Arginine biosynthesis
Taurine and hypotaurine metabolism
-Log10 P
b
Gluconeogenesis
Pyruvate metabolism
Glutathione
Taurine/Hypotaurine
TCA
Pentose phosphate pathway
Synthesis/degradation ketone bodies
Linolenic acid metabolism
-Log10p
Pathway score
c
FAO
Acetyl-CoA
TCA cycle
Citrate
Cis-Aconitate
Aco2
Isocitrate
Mdh1
Oxaloacetate
Malate
Oxalosuccinate
Pck2
PEP
Pyruvate metabolism
Fumarate
2-Oxoglutarate
Sdhc
Succinate
Succinyl-CoA
S-succinyl dihydrolipoamide-E
3-Carboxy 1-hydroxypropyl ThPP
Oxidative phosphorylation/ETC
Cysteine/ methionine metabolism
Aspartate/ glutamate metabolism
Cystine
NADPH
Glutamate
Cysteine
Csad
GSH
Chac1
Taurine Hypotaurine
NADPH
Gpx1/4/7
GSSG
5-Oxoproline
d
Positive mode
p53
N1IC
PC2 (30.7%)
PC1 (55.2%)
Negative mode
PC2 (19.3%)
PC1 (66.6%)
e
Diacylglycerophosphocholines
1-alkyl,2-acylglycerophosphocholines
Contains ether-bond
Glycerophosphocholines
Neutral intrinsic curvature
Endoplasmic reticulum
Headgroup with positive charge/zwitter-ion
Membrane component
C18:0
Headgroup with neutral charge
Glycerolipids
Lipid droplet
Lipid storage
Triacylglycerols
Enriched in p53
Enriched in N1IC
-Log10 P

Appendix Figure S4 (Associated with Figure 3): Differences in energetic metabolism between N1ICand p53 cells

a. Bar plot demonstrating pixel fluorescence intensity ± SEM after quantification of mitoRED staining demonstrating decreased membrane potential in the N1IC model, n=21 p53 cells; n=18 N1IC cells. P-value calculated by Welch's t-test.
b. Quantification of different Seahorse parameters demonstrating higher ATP production, higher respiratory capacity, and higher basal and maximal respiration in p53 tumor cells. Bar graph depicting means ± SEM. Normalized by protein quantification. Data from n=2 independent cell lines per model, n=10 replicates per condition.
c. Seahorse ECAR analysis suggesting increased glycolysis in p53 cells accounting for the higher OCR. Each point represents mean, error bars represent SEM of n=10 independent replicates with n=2 independent cell lines per model.
d. Time course of Seahorse analysis of a p53/N1IC tumor cell pair with or without cysteine/methionine deprivation. Each point represents mean, errors bars represent SEM calculated from n=10 replicates per condition. Normalized by protein quantification.
e. Quantification of Seahorse parameters demonstrating decreased maximal respiration after cysteine/methionine deprivation in p53 cells with no impact on N1IC cells. Normalized by protein quantification. Mean ± SEM from n=5 replicates. b, e: Q-values calculated by two-way ANOVA correcting for multiple comparisons by controlling the FDR using the 2-stage linear step-up procedure of Benjamini, Krieger, and Yekutieli.

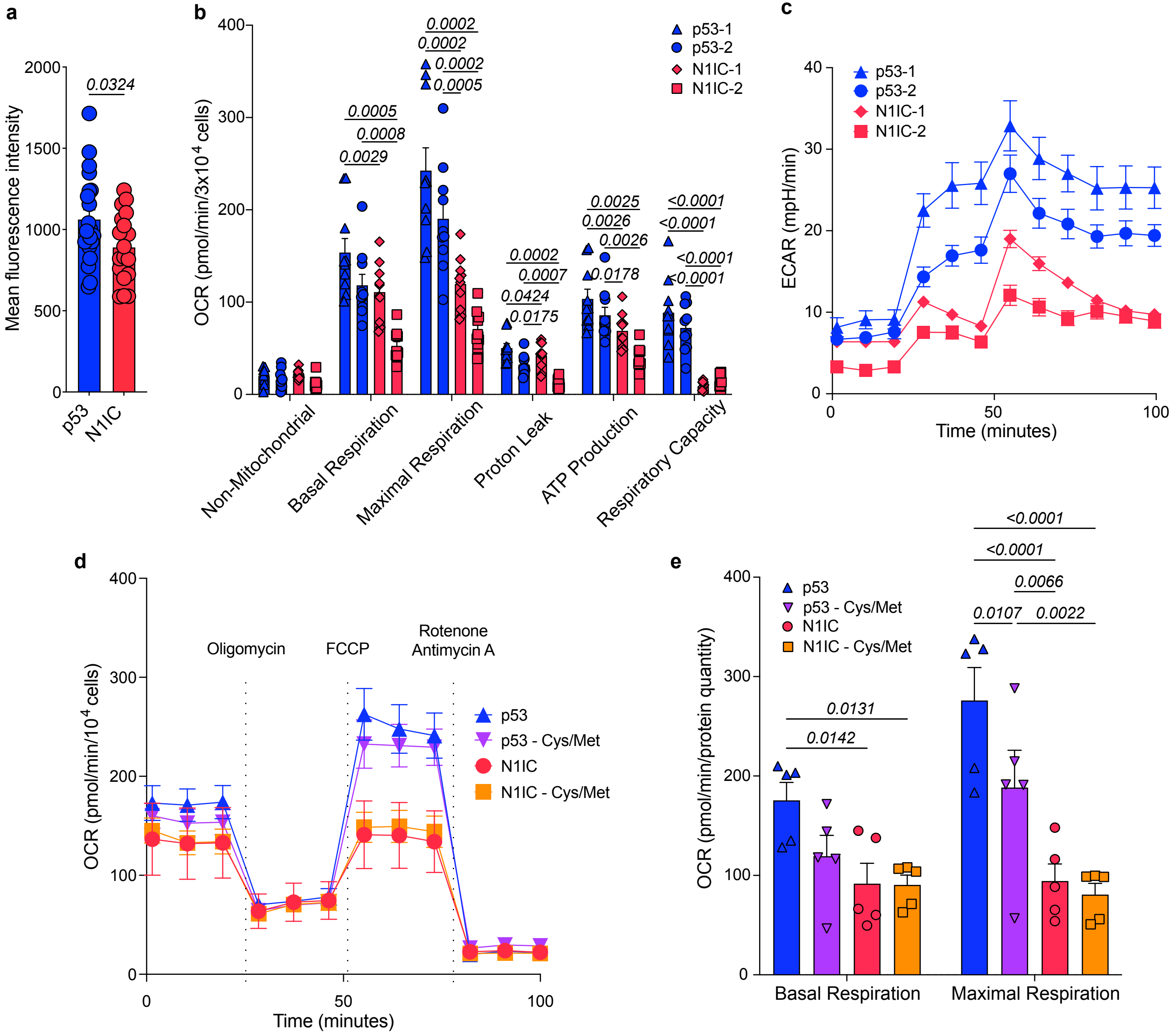
a
Mean fluorescence intensity
0.0324
p53
N1IC
b
OCR (pmol/min/3x10^4 cells)
p53-1
p53-2
N1IC-1
N1IC-2
Non-Mitochondrial
Basal Respiration
Maximal Respiration
Proton Leak
ATP Production
Respiratory Capacity
c
ECAR (mpH/min)
Time (minutes)
d
OCR (pmol/min/10^4 cells)
Oligomycin
FCCP
Rotenone
Antimycin A
p53
p53 - Cys/Met
N1IC
N1IC - Cys/Met
Time (minutes)
e
OCR (pmol/min/protein quantity)
Basal Respiration
Maximal Respiration

Appendix Figure S5 (Associated with Figure 4): Increased sensitivity to Gpx4-driven ferroptosis of N1IC cells

a. Dose response curves in a N1IC cell line treated with RSL3 in combination with ferroptosis, apoptosis and necroptosis inhibitors. Only the ferroptosis inhibitor Ferrostatin (Fer-1, 2 μm) induces partial rescue, while Z-VAD-FKM (50 μm) and Necrostatin-1 (Nec1, 10 μm) have no significant effect, demonstrating that RSL3 induces cell death via ferroptosis-specific mechanisms. Error bars represent SEM from n=3 replicates.
b. Viability with ferroptosis, necroptosis and apoptosis inhibitors alone in the same N1IC cell line. Error bars represent mean $\pm$ SEM from n=5 replicates. P-values calculated by ordinary ANOVA.
c. Live imaging with BODIPY-C11 stains demonstrating time course of 500 nM RSL3-induced ferroptosis in N1IC cell line. The 510/590 ratio, marker of increased lipid peroxidation secondary to ferroptosis, increases 10 to 25 minutes after drug treatment. Scale bar, 50 µm.
d. Dose response curve for ML162, 24-hour treatment, normalized to DMSO. AUC without/with Ferrostatin, n=3 p53/n=2 N1IC cell lines. Error bars represent SEM.
e. Dose response curves for ML210, 48-hour treatment, normalized to DMSO. AUC without/with Ferrostatin, n=2 p53/n=2 N1IC cell lines. Error bars represent SEM. Q-values in d, e calculated by ANOVA with FDR correction via Benjamini, Krieger, and Yekutieli.
f. Western blot of GPX4 in a N1IC cell line demonstrating effective knockdown 48 hours after siGPX4 transfection with no effect of Ferrostatin-1 on GPX4 protein levels. Actin used as loading control. Right: quantification of GPX4 after transfection with GPX4 siRNA, data pooled from n=3 independent experiments. P-values calculated by ordinary ANOVA.
g. Ridge plot demonstrating increased lipid peroxidation as measured by BODIPY-C11 flow cytometry in a p53 cell line transfected for 48 hours with GPX4 siRNA compared to control siRNA or with addition of Ferrostatin-1.
h. Representative microscopic images of a N1IC and p53 cell line transfected for 48 hours with control or GPX4 siRNA with or without Ferrostatin-1. Quantification provided in **Figure 4d.**

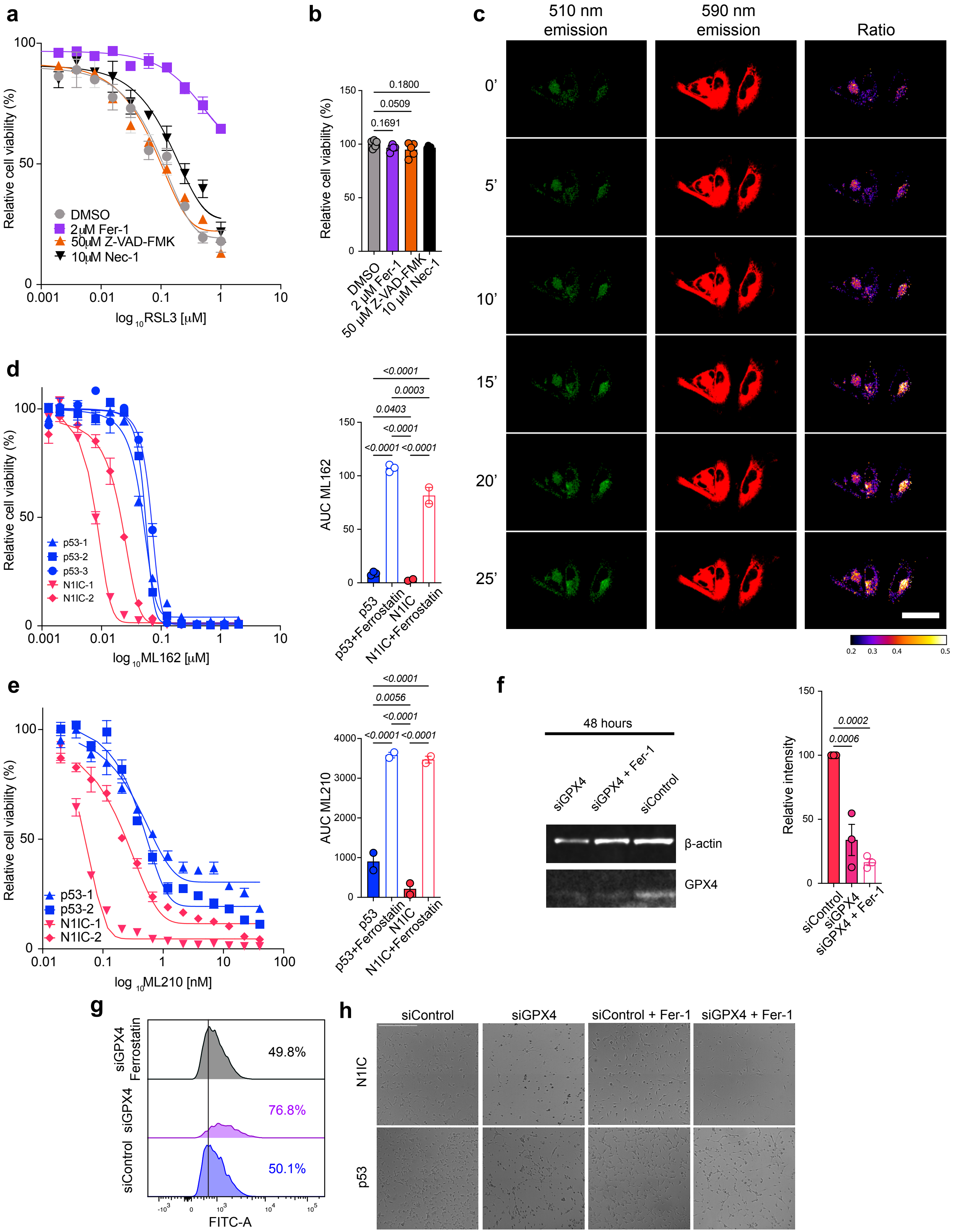

a
Relative cell viability (%)
DMSO
2μM Fer-1
50μM Z-VAD-FMK
10μM Nec-1
log 10RSL3 [μM]
b
Relative cell viability (%)
0.1800
0.0509
0.1691
DMSO
2 μM Fer-1
50 μM Z-VAD-FMK
10 μM Nec-1
c
510 nm emission
590 nm emission
Ratio
0'
5'
10'
15'
20'
25'
d
Relative cell viability (%)
p53-1
p53-2
p53-3
N1IC-1
N1IC-2
log 10ML162 [μM]
AUC ML162
<0.0001
0.0003
0.0403
<0.0001
<0.0001 <0.0001
p53
p53+Ferrostatin
N1IC
N1IC+Ferrostatin
e
Relative cell viability (%)
p53-1
p53-2
N1IC-1
N1IC-2
log 10ML210 [nM]
AUC ML210
<0.0001
0.0056
<0.0001
<0.0001 <0.0001
p53
p53+Ferrostatin
N1IC
N1IC+Ferrostatin
f
48 hours
siGPX4
siGPX4 + Fer-1
siControl
β-actin
GPX4
Relative intensity
0.0002
0.0006
siControl
siGPX4
siGPX4 + Fer-1
g
siGPX4 Ferrostatin
siGPX4
siControl
49.8%
76.8%
50.1%
FITC-A
h
siControl
siGPX4
siControl + Fer-1
siGPX4 + Fer-1
N1IC
p53

Appendix Figure S6: Lack of response to Slc7a11 inhibition in murine glioma models.

a. Dose response curves for the Slc7a11 inhibitor IKE, 24-hour treatment, normalized to DMSO. Error bars represent SEM from n=3 replicates.
b. AUC without and with Ferrostatin, n=3 p53/n=2 N1IC cell lines. Error bars represent SEM. Q-values in d, e calculated by ANOVA with FDR correction via Benjamini, Krieger, and Yekutieli.
c. PCR ΔΔCT ± SEM in two independent p53 and N1IC cell lines for the Slc7a11 transporter demonstrating marginal differences at the transcriptomic level between models. Data pooled from n=3 independent experiments. P-values calculated by one-way ANOVA.
d. Representative Western blot demonstrating presence of the Slc7a11 transporter in two independent p53 and N1IC murine cell cultures. Note there are no significant differences in Slc7a11 protein levels between models. Actin used as loading control.
e. Bar plot demonstrating mean GSH fluorescence levels ± SEM and mean GSH/GSSG ratio ± SEM after treatment with DMSO vehicle (red) or 100 and 1000 nM IKE (pink) in a N1IC cell line demonstrating decrease in GSH fluorescence but no significant difference in GSH/GSSG ratio after inhibition of Slc7a11. Data pooled from n=2 independent experiments. P-values calculated by one-way ANOVA.

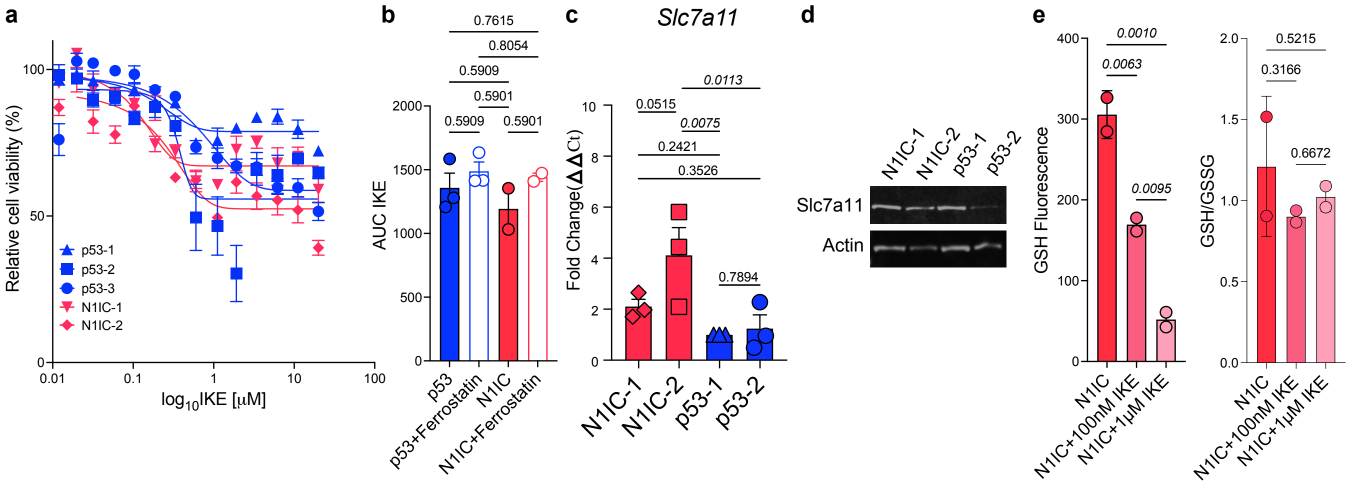

a
Relative cell viability (%)
log10IKE [μM]
p53-1
p53-2
p53-3
N1IC-1
N1IC-2
b
AUC IKE
0.7615
0.8054
0.5909
0.5901
0.5909
0.5901
p53
p53+Ferrostatin
N1IC
N1IC+Ferrostatin
c
Slc7a11
Fold Change(ΔΔCt)
0.0113
0.0515
0.0075
0.2421
0.3526
0.7894
N1IC-1
N1IC-2
p53-1
p53-2
d
N1IC-1
N1IC-2
p53-1
p53-2
Slc7a11
Actin
e
GSH Fluorescence
0.0010
0.0063
0.0095
N1IC
N1IC+100nM IKE
N1IC+1μM IKE
GSH/GSSG
0.5215
0.3166
0.6672
N1IC
N1IC+100nM IKE
N1IC+1μM IKE

Appendix Figure S7 (Associated with Figure 5): Impact of ETC modulation and ROS scavengers on lipid peroxidation and ROS production

a-d. Response to mitochondrial ETC inhibitors demonstrating no difference in response to complex I inhibitor IACS (a), complex III inhibitor Antimycin (b) or complex V/ATP synthase inhibitor Oligomycin (c) and minimal differential sensitivity to uncoupling agent FCCP (d) between p53/N1IC cells. Glioma cells are most sensitive to complex III inhibition. n=6 replicates per condition, n=4 replicates for Oligomycin. P-values calculated via unpaired t test.

e. Violin plots depicting JC-1 ratio in p53 and N1IC cells treated with FCCP demonstrating decrease in mitochondrial membrane potential as expected after uncoupling of oxidative phosphorylation.

f. Dose response curves in N1IC cells treated with DMSO followed by RSL3 (red), FCCP followed by RSL3 (light blue) and IACS followed by RSL3 (dark green) demonstrating the need for effective ROS production at complex I to effectively initiate ferroptosis. Error bars represent SEM from n=3 replicates.

g. Bar plots depicting mean ROS $\pm$ SEM after two different doses of the TSPO inhibitor PK11195 demonstrating no impact on total ROS production with TSPOi treatment. Data pooled from n=3 independent experiments. P-values calculated by unpaired ANOVA.

h. Violin plots depicting JC-1 ratio in a N1IC cell line treated with DMSO vehicle or 10 μM PK11195 demonstrating marginal decrease in mitochondrial membrane potential.

i-k. Bar graphs depicting mean $\pm$ SEM MitoCLOX, BODIPY-C11 and H2DCFDA in a N1IC cell line treated with the ER-targeted ROS scavenger Ferrostatin-1 or the iron chelator DFO. Both DFO and Ferrostatin-1 inhibit ferroptosis but have no effect on total ROS. DFO but not Ferrostatin-1 decreases mitochondrial lipid peroxidation. P-values calculated by unpaired t-test. Data pooled from n=4 (DFO) and n=5 (Ferrostatin) independent experiments.

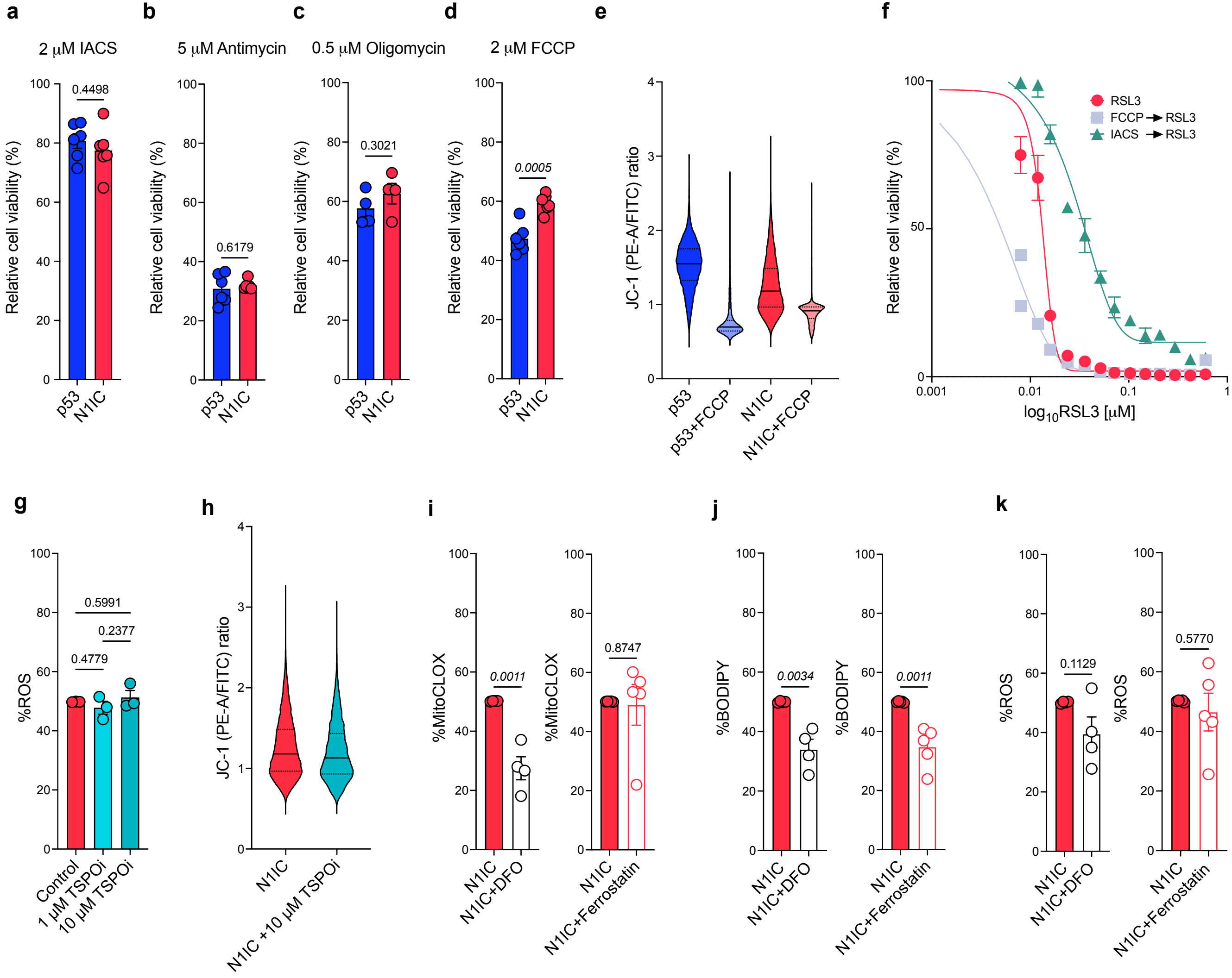
a
b
c
d
e
f
2 μM IACS
5 μM Antimycin
0.5 μM Oligomycin
2 μM FCCP
Relative cell viability (%)
0.4498
0.6179
0.3021
0.0005
p53
N1IC
JC-1 (PE-A/FITC) ratio
p53+FCCP
N1IC+FCCP
RSL3
FCCP → RSL3
IACS → RSL3
log10RSL3 [μM]
g
h
i
j
k
%ROS
0.5991
0.2377
0.4779
Control
1 μM TSPOi
10 μM TSPOi
N1IC +10 μM TSPOi
%MitoCLOX
0.0011
0.8747
N1IC+DFO
N1IC+Ferrostatin
%BODIPY
0.0034
0.0011
0.1129
0.5770

Appendix Figure S8: N1IC-like tumor populations in treatment-naïve and post-treatment human GBM

a. UMAP embedding of scRNA-seq data from n=8 GBM patient samples described in (Yuan *et al*, 2018) colored by sample (left) and by proliferation status and cell state (right)
b. Same as a. but colored by expression of TSPO
c. Violin plots of TSPO expression in proliferating vs quiescent transformed populations in human GBM samples. P-value calculated by Welch's t-test.
d. Heatmap of Spearman correlation index between the AUCell gene signatures generated from the Notch murine model (N1IC_up and N1IC_down) and Neftel cell state using the scRNA-seq data in panels a-b. Note the clustering of the "N1IC_up" signature with the AC/MES signatures. The "N1IC_down" signature clusters with the NPC/OPC and proliferation signatures.
e. Left: Bar plot demonstrating enrichment in the "N1IC_up" signature in 4 out of 5 patients undergoing chronic intratumoral delivery of topotecan in a recurrent GBM cohort (Spinazzi *et al*, 2022). Right: GSEA of the "N1IC_up" signature in all 5 patients combined.
f. Heatmap of Spearman correlation between cell states and metabolic gene ontologies using the same scRNA-seq dataset used in panels a-b. Note divergent programs in the AC and NPC cell states, including upregulated mitochondrial programs in AC and enriched amino acid programs in NPC. Full list provided in **Dataset EV5**.

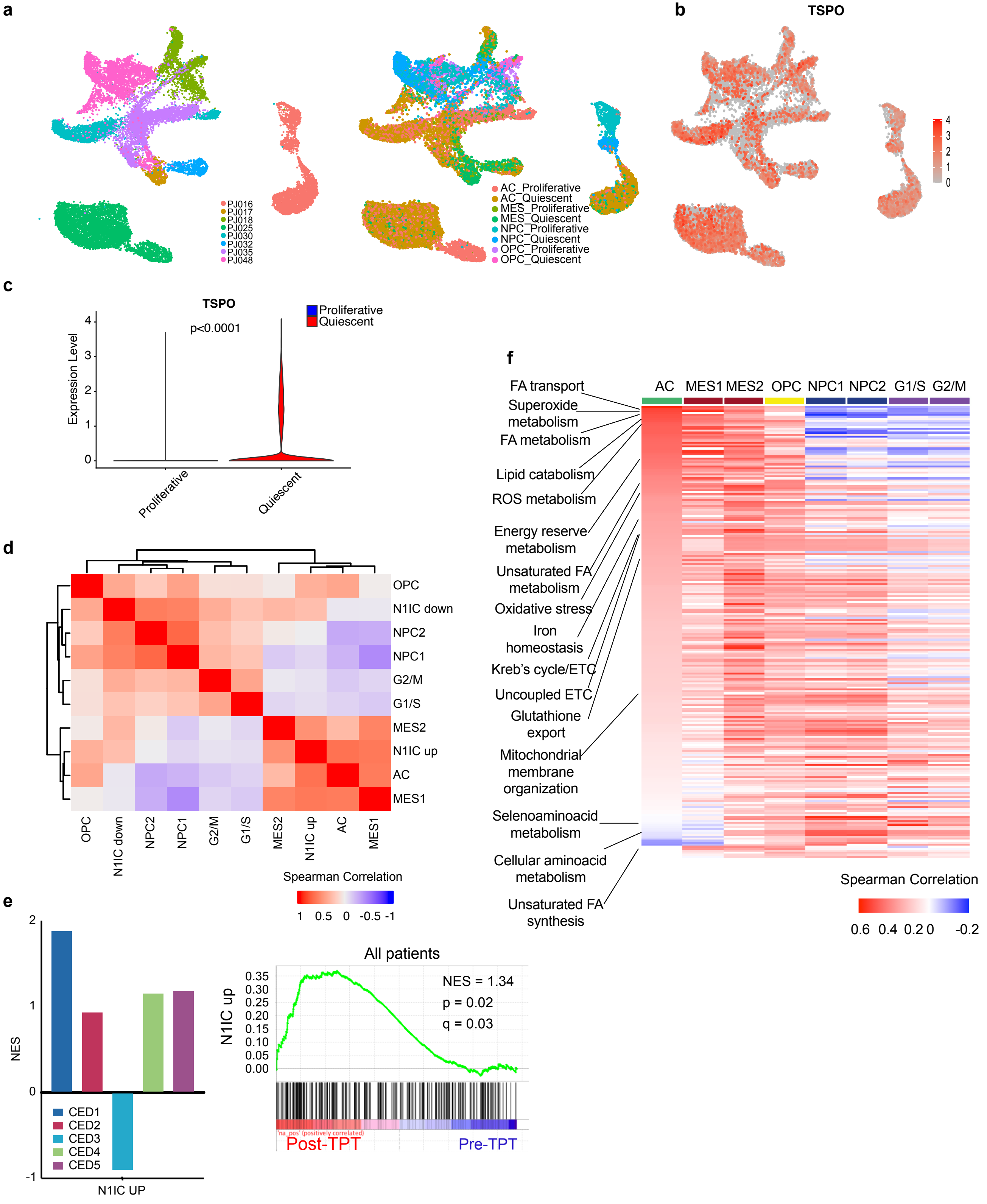

a
PJ016
PJ017
PJ018
PJ025
PJ030
PJ032
PJ035
PJ048
AC_Proliferative
AC_Quiescent
MES_Proliferative
MES_Quiescent
NPC_Proliferative
NPC_Quiescent
OPC_Proliferative
OPC_Quiescent
b
TSPO
4
3
2
1
0
c
TSPO
p<0.0001
Proliferative
Quiescent
Expression Level
Proliferative
Quiescent
d
OPC
N1IC down
NPC2
NPC1
G2/M
G1/S
MES2
N1IC up
AC
MES1
Spearman Correlation
1 0.5 0 -0.5 -1
e
NES
CED1
CED2
CED3
CED4
CED5
N1IC UP
All patients
N1IC up
NES = 1.34
p = 0.02
q = 0.03
Post-TPT
Pre-TPT
f
FA transport
Superoxide metabolism
FA metabolism
Lipid catabolism
ROS metabolism
Energy reserve metabolism
Unsaturated FA metabolism
Oxidative stress
Iron homeostasis
Kreb's cycle/ETC
Uncoupled ETC
Glutathione export
Mitochondrial membrane organization
Selenoaminoacid metabolism
Cellular aminoacid metabolism
Unsaturated FA synthesis
AC
MES1
MES2
OPC
NPC1
NPC2
G1/S
G2/M
Spearman Correlation
0.6 0.4 0.2 0 -0.2

Appendix Figure S9: Effects of Notch activation are context dependent in patient-derived transformed cell lines

a. Heatmap of Spearman correlation index between glioma cell state scores and Notch signaling using bulk RNA-seq data from glioma cell lines in the Cancer Cell Line Encyclopedia (CCLE) dataset demonstrating no significant correlation between Notch activation and cell state in vitro. Note the positive correlations between all cell state scores demonstrating poor preservation of cell identity *in vitro*. P-values provided in **Dataset EV7**.
b. Heatmap of Notch signaling and TSPO expression based on bulk RNA-seq glioma cell data from CCLE demonstrating poor correlation between Notch status and ferroptosis susceptibility but anticorrelation between RSL3 AUC and TSPO expression. P-values in **Dataset EV7**.
c. Bar plots depicting mean $\pm$ SEM for RSL3 AUC, GSH/GSSG ratio, $\log_{10}$NAD and $\log_{10}$NADH relative abundance in Notch low (blue) and Notch high (red) (Methods) glioma cell lines.
d. Volcano plot of differentially expressed genes in control GFP compared to N1IC overexpressing TS543 cells. N1IC upregulated genes in red. Note upregulated canonical Notch target genes HES5, HEY1, HEY2, HEYL and downregulated NPC/OPC genes ASCL1, OLIG1, OLIG2 after Notch overexpression. Inset: GSEA using glioma cell state gene signatures demonstrating enrichment in the MES1 gene signature with Notch activation. Only signatures with FDR<0.05 depicted. Also refer to **Table EV2** for complete list of differentially expressed genes.
e. Bar graph depicting significant metabolic gene ontologies in control GFP vs N1IC overexpressing TS543 cells. Blue enriched in GFP, red enriched in N1IC overexpression. NES – normalized enrichment score. Full list of transcriptional metabolic programs provided in **Table EV2**. Only gene sets with nominal p value<0.05 are shown.
f. qRT-PCR of direct Notch downstream targets and NPC markers demonstrating downregulation of *HEY1, HEY2, HEYL* in TS543 dnMAML without effect on OLIG2 or SOX2 expression. Data pooled from n=3 independent experiments. P-values via unpaired t test.
g. Dose response curve in control GFP and dnMAML TS543 treated with RSL3 or DMSO vehicle with or without Ferrostatin 1 demonstrating no differential response with Notch genetic inhibition. Error bars represent SEM from n=3 replicates.
h. AUC for RSL3 in GFP vs dnMAML TS543. Data pooled from n=3 independent drug screens, P-value calculated by Welch's t-test.
i. Dose response curve in control GFP and dnMAML TS543 treated with IKE or DMSO vehicle with or without Ferrostatin 1 demonstrating no differential response with Notch genetic inhibition. Error bars represent SEM from n=3 replicates.
j. AUC for IKE in GFP vs dnMAML TS543. Data pooled from n=3 independent drug screens, P-value calculated by Welch's t-test.

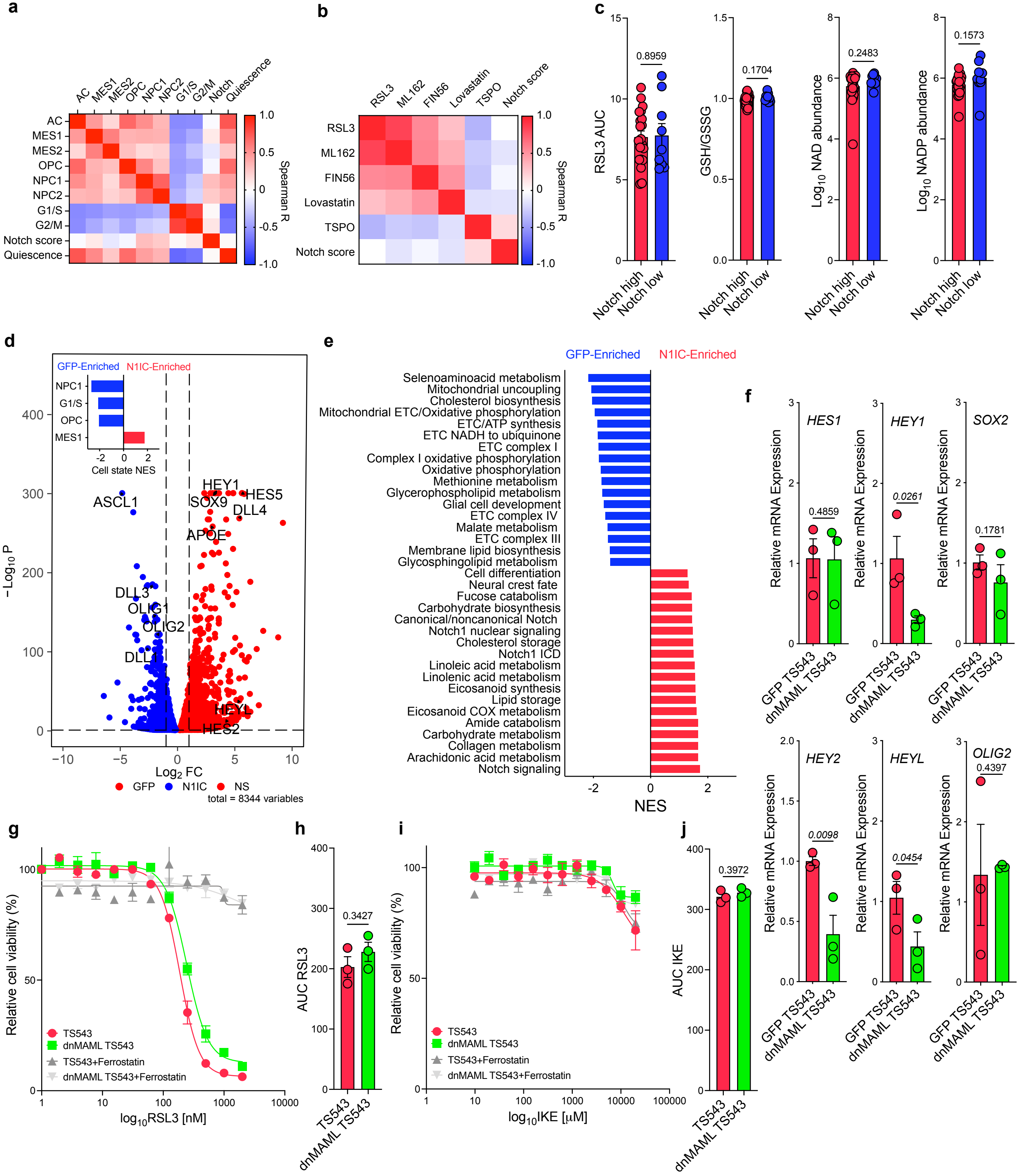

a
AC
MES1
MES2
OPC
NPC1
NPC2
G1/S
G2/M
Notch score
Quiescence
Spearman R
1.0
0.5
0
-0.5
-1.0
b
RSL3
ML162
FIN56
Lovastatin
TSPO
Notch score
c
RSL3 AUC
GSH/GSSG
Log10 NAD abundance
Log10 NADP abundance
0.8959
0.1704
0.2483
0.1573
Notch high
Notch low
d
GFP-Enriched
N1IC-Enriched
NPC1
G1/S
OPC
MES1
Cell state NES
−Log10 P
Log2 FC
ASCL1
HEY1
SOX9
HES5
DLL4
APOE
DLL3
OLIG1
OLIG2
DLL1
HEYL
HES2
GFP
N1IC
NS
total = 8344 variables
e
GFP-Enriched
N1IC-Enriched
Selenoaminoacid metabolism
Mitochondrial uncoupling
Cholesterol biosynthesis
Mitochondrial ETC/Oxidative phosphorylation
ETC/ATP synthesis
ETC NADH to ubiquinone
ETC complex I
Complex I oxidative phosphorylation
Oxidative phosphorylation
Methionine metabolism
Glycerophospholipid metabolism
Glial cell development
ETC complex IV
Malate metabolism
ETC complex III
Membrane lipid biosynthesis
Glycosphingolipid metabolism
Cell differentiation
Neural crest fate
Fucose catabolism
Carbohydrate biosynthesis
Canonical/noncanonical Notch
Notch1 nuclear signaling
Cholesterol storage
Notch1 ICD
Linoleic acid metabolism
Linolenic acid metabolism
Eicosanoid synthesis
Lipid storage
Eicosanoid COX metabolism
Amide catabolism
Carbohydrate metabolism
Collagen metabolism
Arachidonic acid metabolism
Notch signaling
NES
f
Relative mRNA Expression
HES1
HEY1
SOX2
HEY2
HEYL
OLIG2
0.4859
0.0261
0.1781
0.0098
0.0454
0.4397
GFP TS543
dnMAML TS543
g
Relative cell viability (%)
log10RSL3 [nM]
TS543
dnMAML TS543
TS543+Ferrostatin
dnMAML TS543+Ferrostatin
h
AUC RSL3
0.3427
TS543
dnMAML TS543
i
Relative cell viability (%)
log10IKE [µM]
TS543
dnMAML TS543
TS543+Ferrostatin
dnMAML TS543+Ferrostatin
j
AUC IKE
0.3972
TS543
dnMAML TS543

Appendix Figure S10: Selection or transformation of the AC-like population in PDX models

a. UMAP embedding of scRNA-seq data from TS543 PDX model at 21 days post-injection colored by replicate.
b. Same as a. but colored by the relative expression of human transcripts indicative of whether the cells are transformed tumor cells (human) or untransformed cells in the brain microenvironment (mouse).
c. UMAP embedding of scRNA-seq data from a after isolating the transformed tumor cells colored by replicate.
d. Same as c. but colored based on unsupervised clustering of the single-cell expression profiles.
e. Heatmap of average gene expression for markers of various GBM cell subsets and states across clusters and replicates.
f. Heatmap of normalized enrichment scores (NES) from GSEA of GBM subset signatures from Neftel et al(Neftel *et al*, 2019) across clusters and replicates.

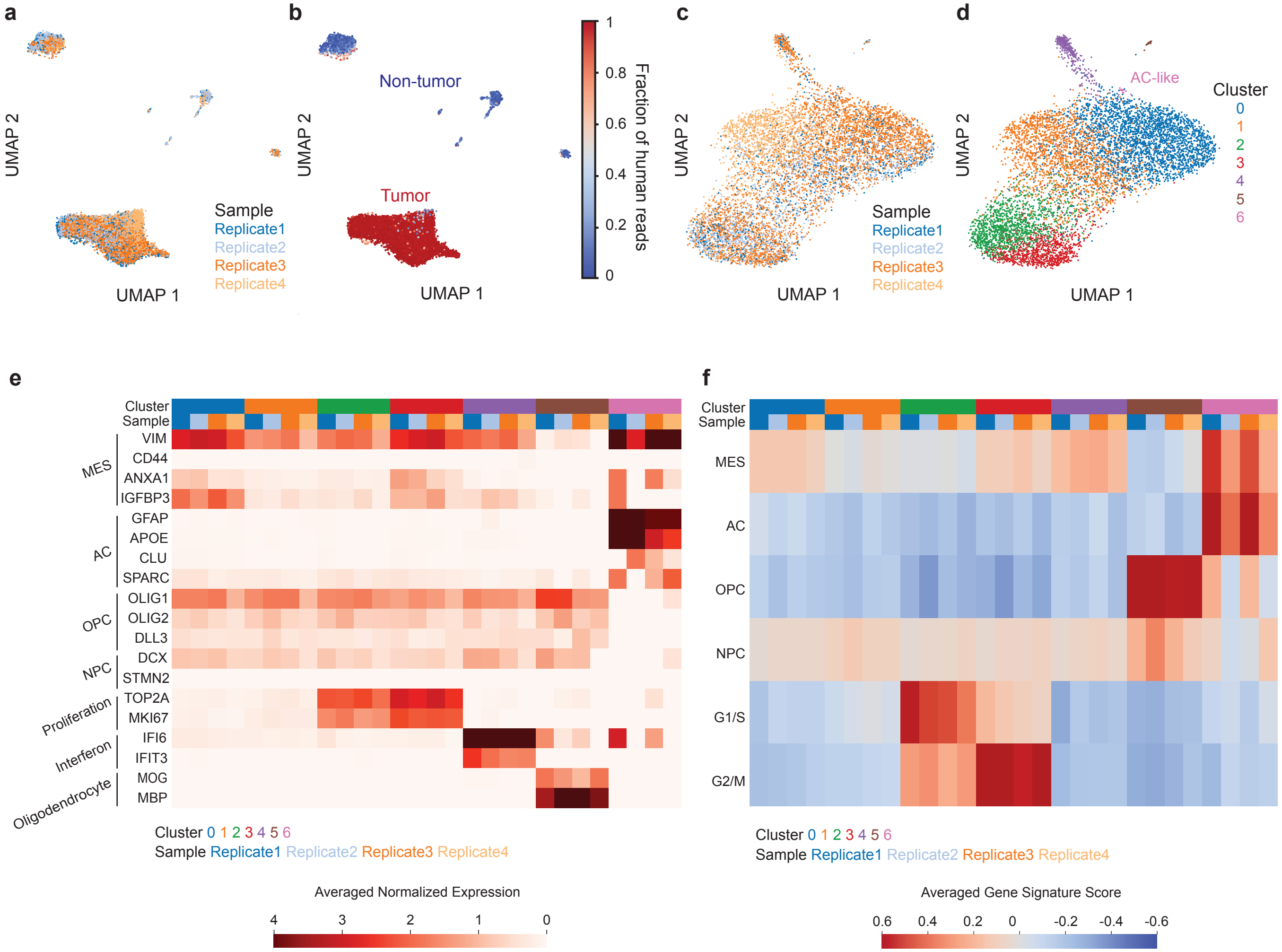

a
UMAP 2
UMAP 1
Sample
Replicate1
Replicate2
Replicate3
Replicate4
b
Non-tumor
Tumor
UMAP 2
UMAP 1
Fraction of human reads
1
0.8
0.6
0.4
0.2
0
c
UMAP 2
UMAP 1
Sample
Replicate1
Replicate2
Replicate3
Replicate4
d
AC-like
Cluster
0
1
2
3
4
5
6
UMAP 2
UMAP 1
e
Cluster
Sample
MES
VIM
CD44
ANXA1
IGFBP3
AC
GFAP
APOE
CLU
SPARC
OPC
OLIG1
OLIG2
DLL3
NPC
DCX
STMN2
Proliferation
TOP2A
MKI67
Interferon
IFI6
IFIT3
Oligodendrocyte
MOG
MBP
Cluster 0 1 2 3 4 5 6
Sample Replicate1 Replicate2 Replicate3 Replicate4
Averaged Normalized Expression
4 3 2 1 0
f
Cluster
Sample
MES
AC
OPC
NPC
G1/S
G2/M
Cluster 0 1 2 3 4 5 6
Sample Replicate1 Replicate2 Replicate3 Replicate4
Averaged Gene Signature Score
0.6 0.4 0.2 0 -0.2 -0.4 -0.6

Appendix Figure S11 (Associated with Figure 7): Identification of transformed populations in slice cultures via chromosome copy number alterations

a. Heatmaps showing the average chromosomal copy number from whole genome sequencing of patient samples used for slice cultures in **Figure 7**.
b. Left: UMAP embeddings of scRNA-seq profiles from TB6181, TB6328, TB6458, TB6528, TB6534 and TB6545 slice cultures colored by the log-ratio of Chr. 2 to Chr. 1 average expression (TB6181) or by the log-ratio of Chr. 7 to Chr. 10 average expression (TB6328, TB6458, TB6528, TB6534 and TB6545). The high ratio (red) indicates malignant transformation. Right: Violin plots showing the distributions of log-ratios of Chr. 2 to Chr. 1 or Chr. 7 to Chr. 10 average expression for each Phenograph cluster identified for TB6181, TB6328, TB6458, TB6528, TB6534 and TB6545 slice cultures.

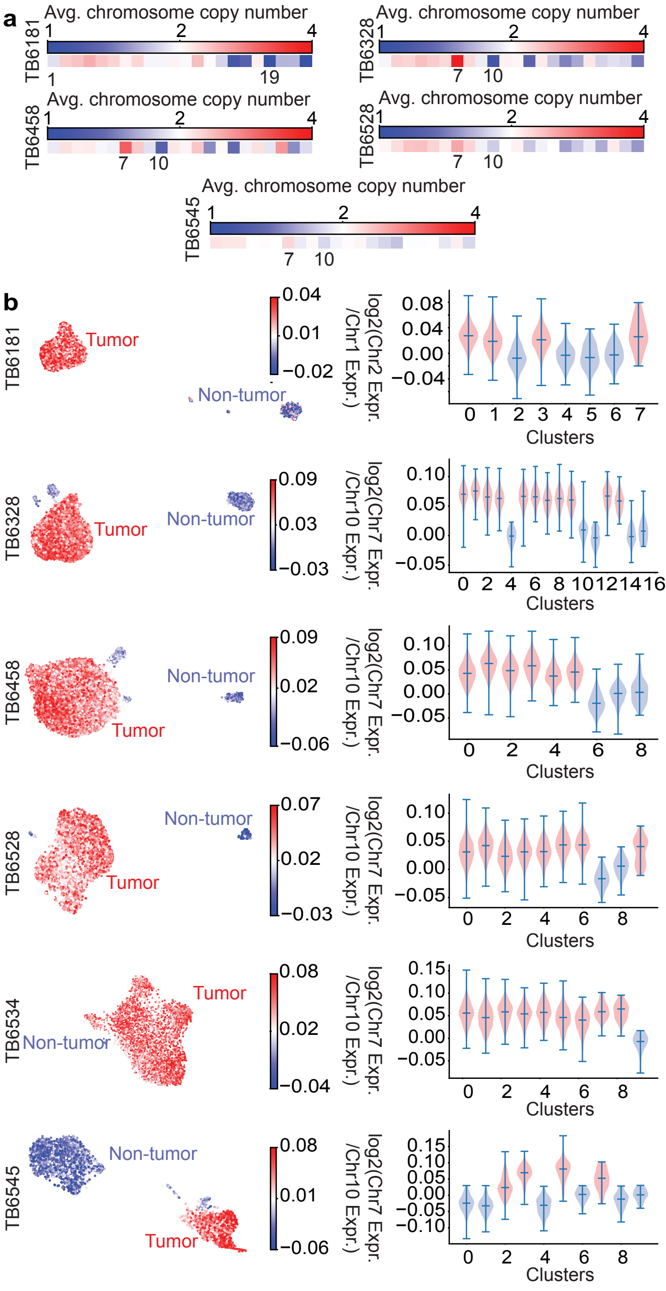

a
TB6181
Avg. chromosome copy number
1
2
4
1
19
TB6328
Avg. chromosome copy number
1
2
4
7
10
TB6458
Avg. chromosome copy number
1
2
4
7
10
TB6528
Avg. chromosome copy number
1
2
4
7
10
TB6545
Avg. chromosome copy number
1
2
4
7
10
b
TB6181
Tumor
Non-tumor
0.04
0.01
−0.02
log2(Chr2 Expr. /Chr1 Expr.)
0.08
0.04
0.00
−0.04
0 1 2 3 4 5 6 7
Clusters
TB6328
Tumor
Non-tumor
0.09
0.03
−0.03
log2(Chr7 Expr. /Chr10 Expr.)
0.10
0.05
0.00
−0.05
0 2 4 6 8 10 12 14 16
Clusters
TB6458
Non-tumor
Tumor
0.09
0.02
−0.06
log2(Chr7 Expr. /Chr10 Expr.)
0.10
0.05
0.00
−0.05
0 2 4 6 8
Clusters
TB6528
Non-tumor
Tumor
0.07
0.02
−0.03
log2(Chr7 Expr. /Chr10 Expr.)
0.10
0.05
0.00
−0.05
0 2 4 6 8
Clusters
TB6534
Tumor
Non-tumor
0.08
0.02
−0.04
log2(Chr7 Expr. /Chr10 Expr.)
0.15
0.10
0.05
0.00
−0.05
0 2 4 6 8
Clusters
TB6545
Non-tumor
Tumor
0.08
0.01
−0.06
log2(Chr7 Expr. /Chr10 Expr.)
0.15
0.10
0.05
0.00
−0.05
−0.10
0 2 4 6 8
Clusters

REFERENCES

Liau BB, Sievers C, Donohue LK, Gillespie SM, Flavahan WA, Miller TE, Venteicher AS, Hebert CH, Carey CD, Rodig SJ *et al* (2017) Adaptive Chromatin Remodeling Drives Glioblastoma Stem Cell Plasticity and Drug Tolerance. *Cell Stem Cell* 20: 233-246 e237

Neftel C, Laffy J, Filbin MG, Hara T, Shore ME, Rahme GJ, Richman AR, Silverbush D, Shaw ML, Hebert CM *et al* (2019) An Integrative Model of Cellular States, Plasticity, and Genetics for Glioblastoma. *Cell* 178: 835-849 e821

Spinazzi EF, Argenziano MG, Upadhyayula PS, Banu MA, Neira JA, Higgins DMO, Wu PB, Pereira B, Mahajan A, Humala N *et al* (2022) Chronic convection-enhanced delivery of topotecan for patients with recurrent glioblastoma: a first-in-patient, single-centre, single-arm, phase 1b trial. *Lancet Oncol* 23: 1409-1418

Yuan J, Levitin HM, Frattini V, Bush EC, Boyett DM, Samanamud J, Ceccarelli M, Dovas A, Zanazzi G, Canoll P *et al* (2018) Single-cell transcriptome analysis of lineage diversity in high-grade glioma. *Genome Med* 10: 57
