# Supplementary figures and images for: A cell state-specific metabolic vulnerability to GPX4-dependent ferroptosis in glioblastoma
Source: EMBO J. 2024 Aug 27;43(20):4492–521. doi: 10.1038/s44318-024-00176-4 (PMC11480389; doi:10.1038/s44318-024-00176-4)

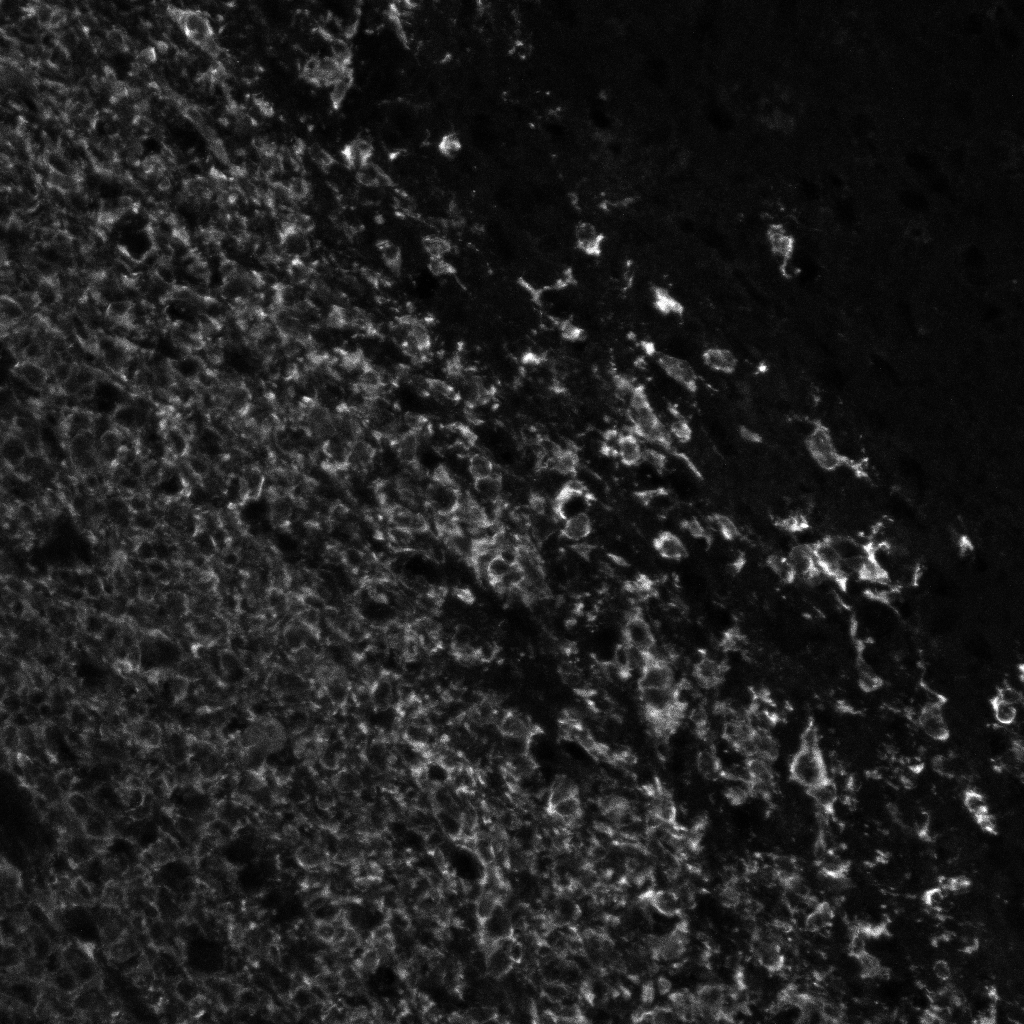

Supplement: Supplementary file 14 — Source data Fig. 1 [file 44318_2024_176_MOESM14_ESM.zip › Figure1/1D/P53_HA.tif]

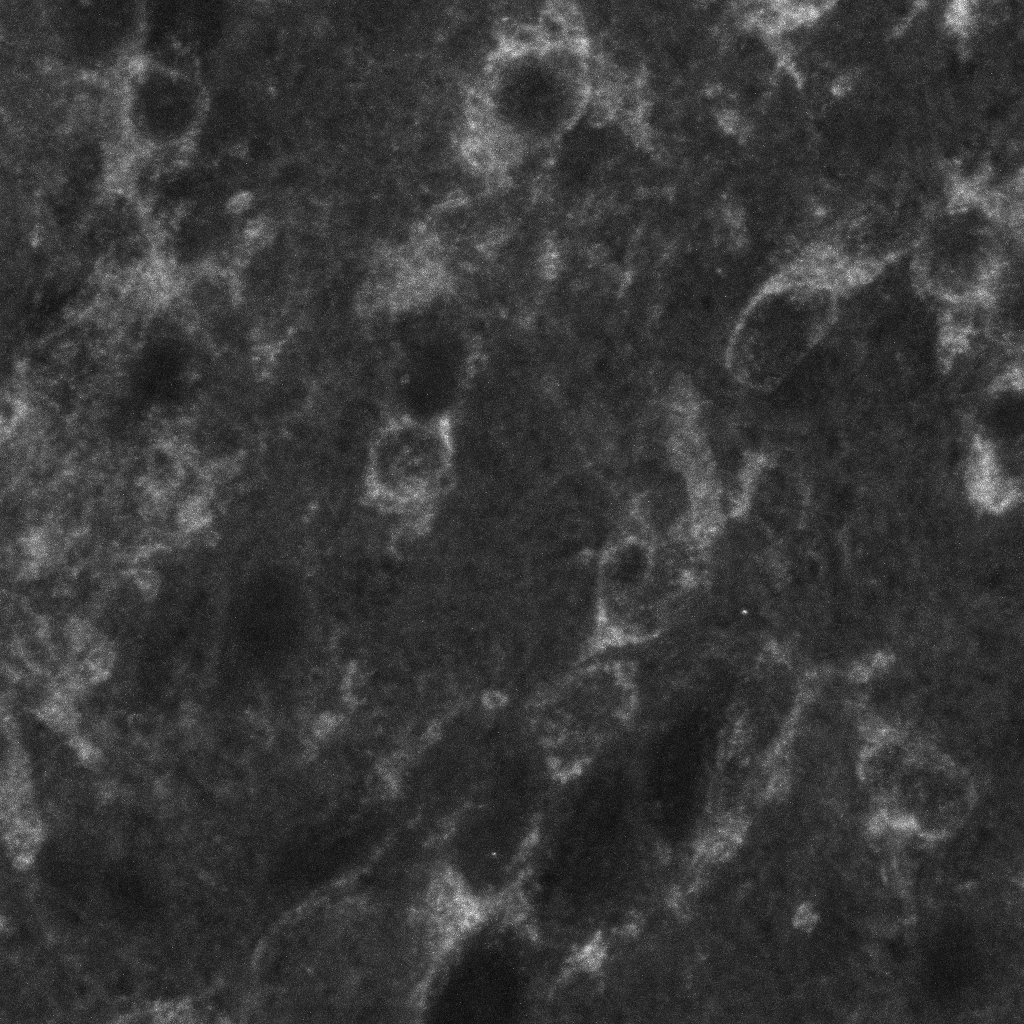

Supplement: Supplementary file 14 — Source data Fig. 1 [file 44318_2024_176_MOESM14_ESM.zip › Figure1/1F/P53_HA.tif]

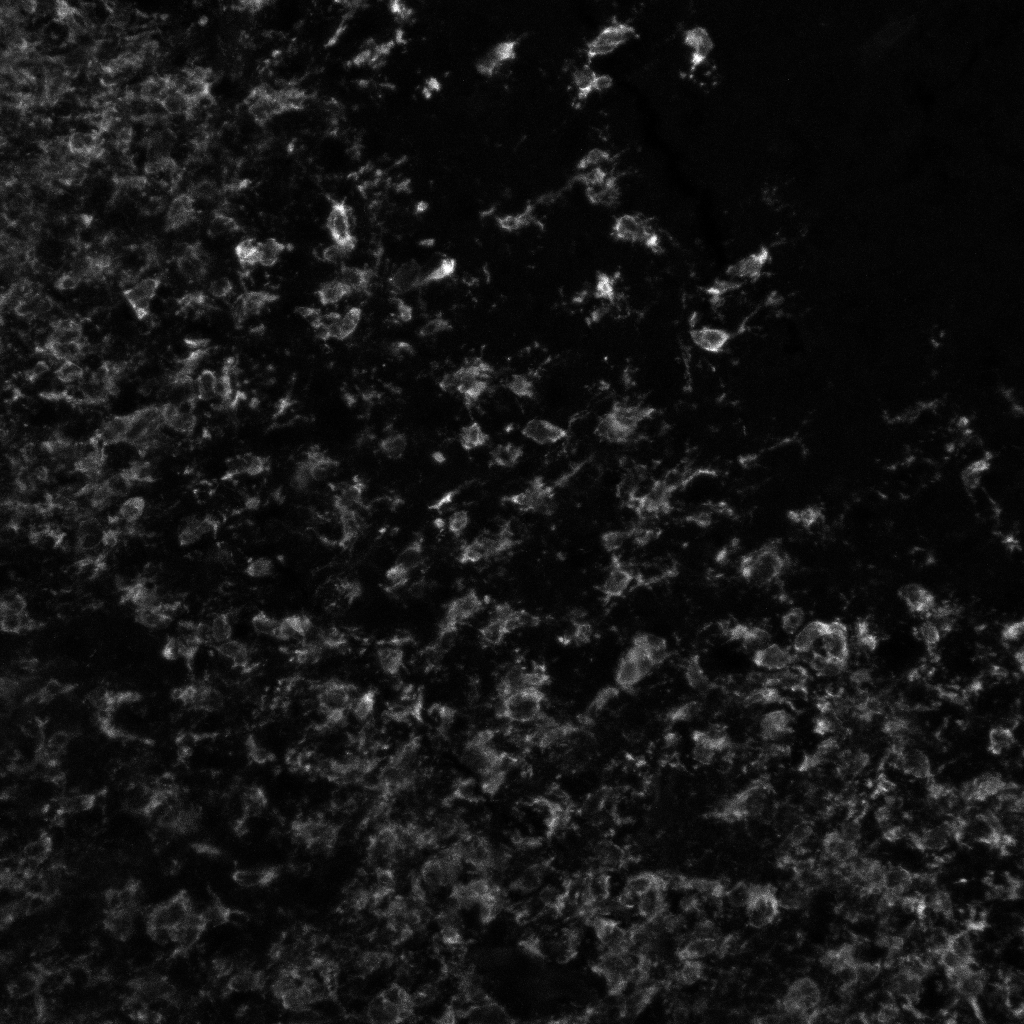

Supplement: Supplementary file 14 — Source data Fig. 1 [file 44318_2024_176_MOESM14_ESM.zip › Figure1/1D/N1IC_HA.tif]

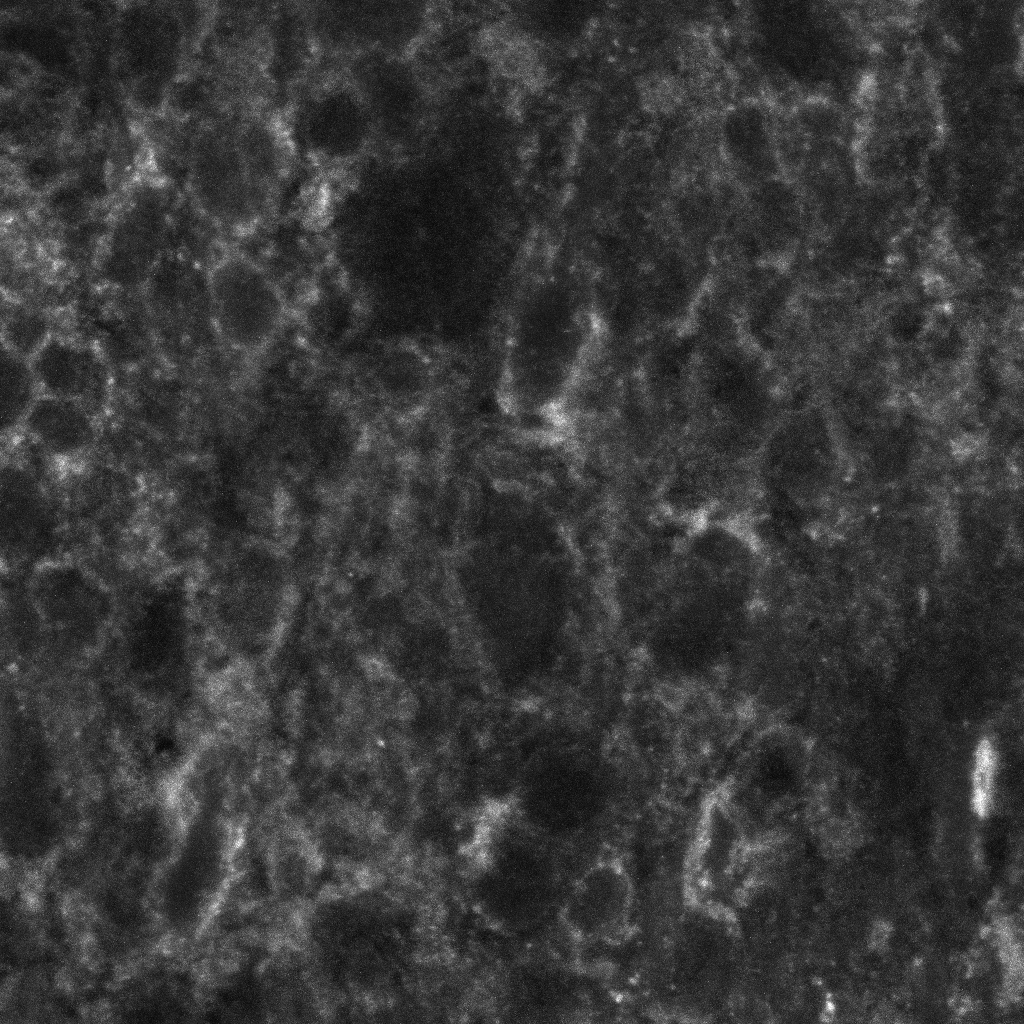

Supplement: Supplementary file 14 — Source data Fig. 1 [file 44318_2024_176_MOESM14_ESM.zip › Figure1/1F/N1IC_HA.tif]

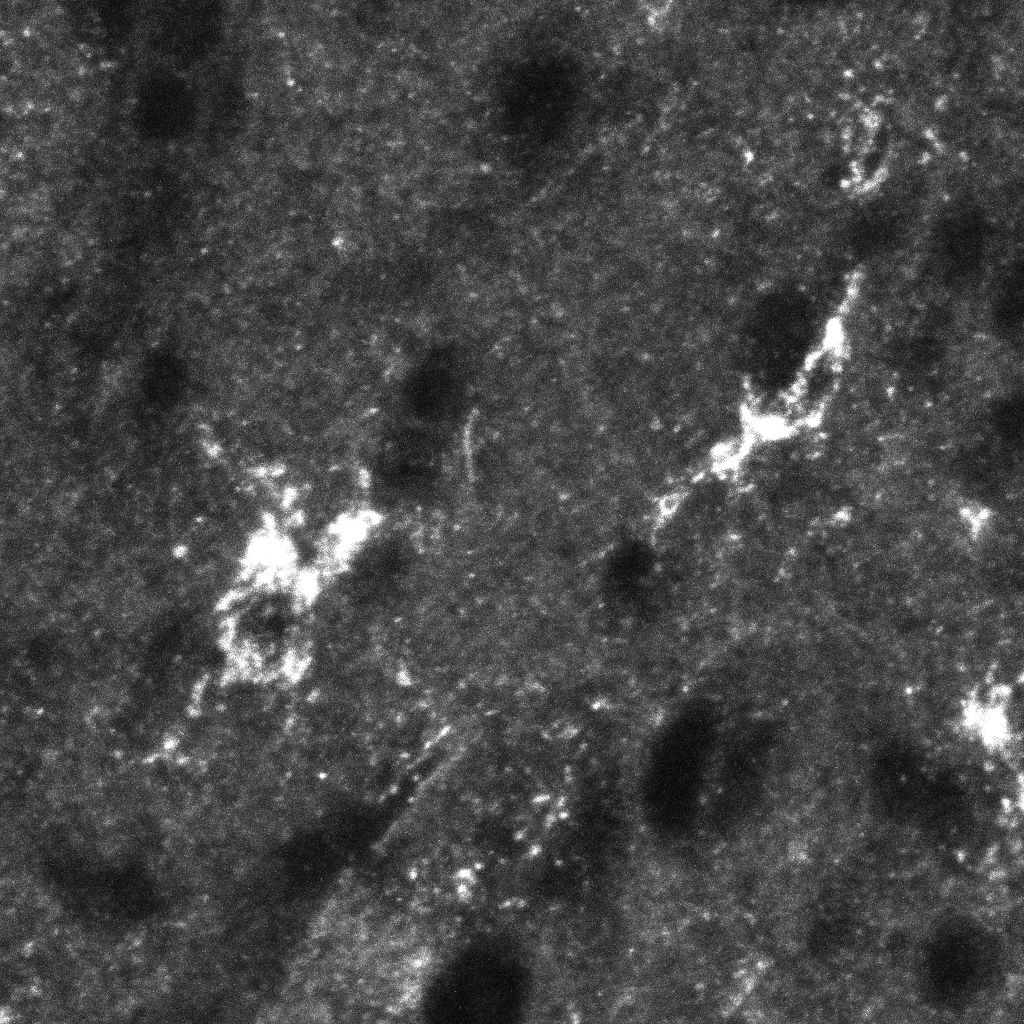

Supplement: Supplementary file 14 — Source data Fig. 1 [file 44318_2024_176_MOESM14_ESM.zip › Figure1/1F/P53_CLU.tif]

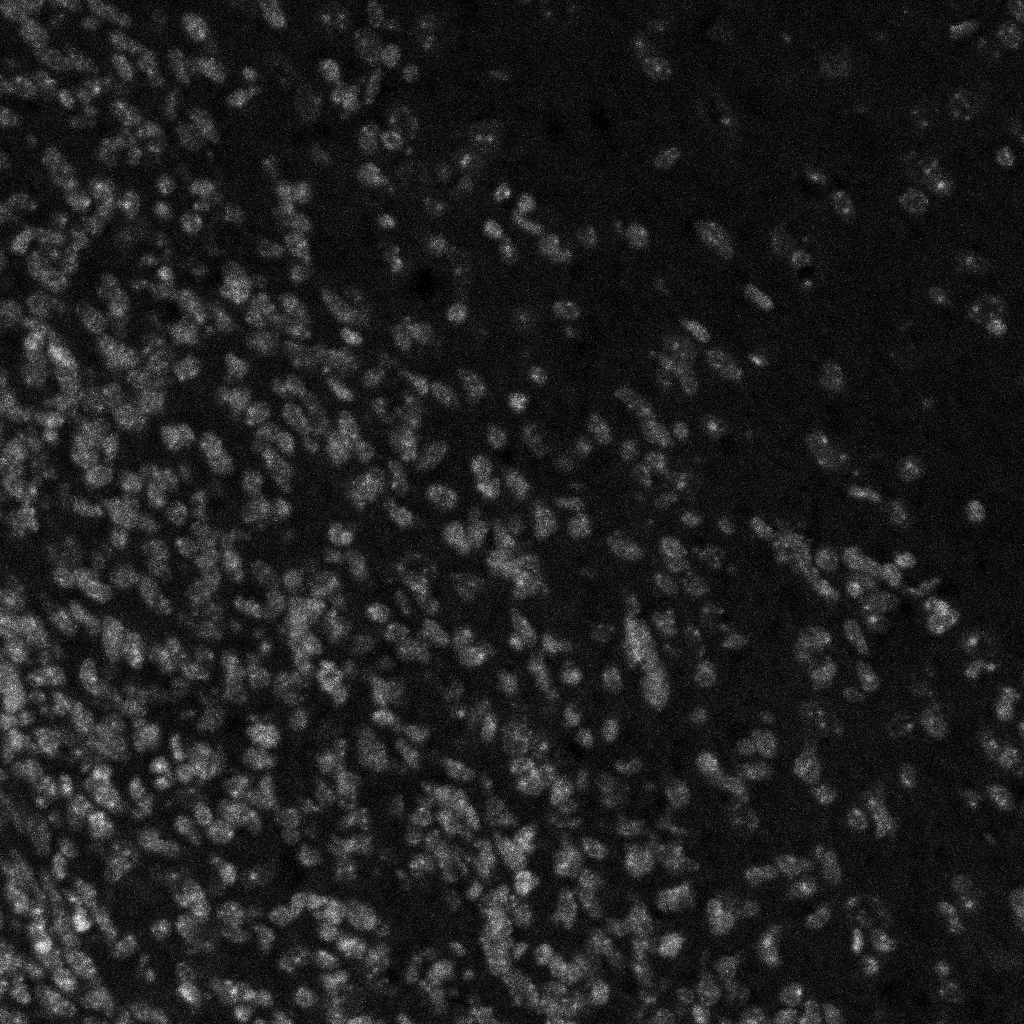

Supplement: Supplementary file 14 — Source data Fig. 1 [file 44318_2024_176_MOESM14_ESM.zip › Figure1/1D/P53_DAPI.tif]

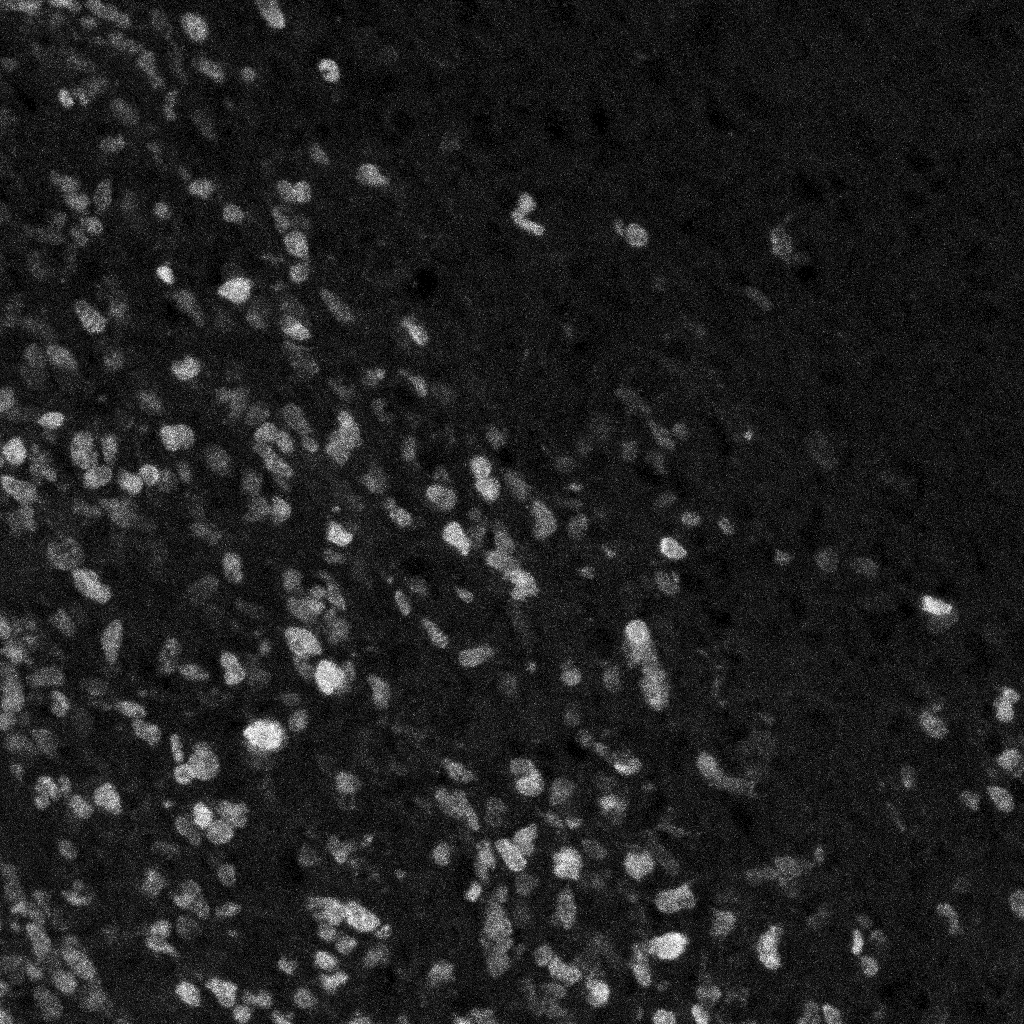

Supplement: Supplementary file 14 — Source data Fig. 1 [file 44318_2024_176_MOESM14_ESM.zip › Figure1/1D/P53_Ki67.tif]

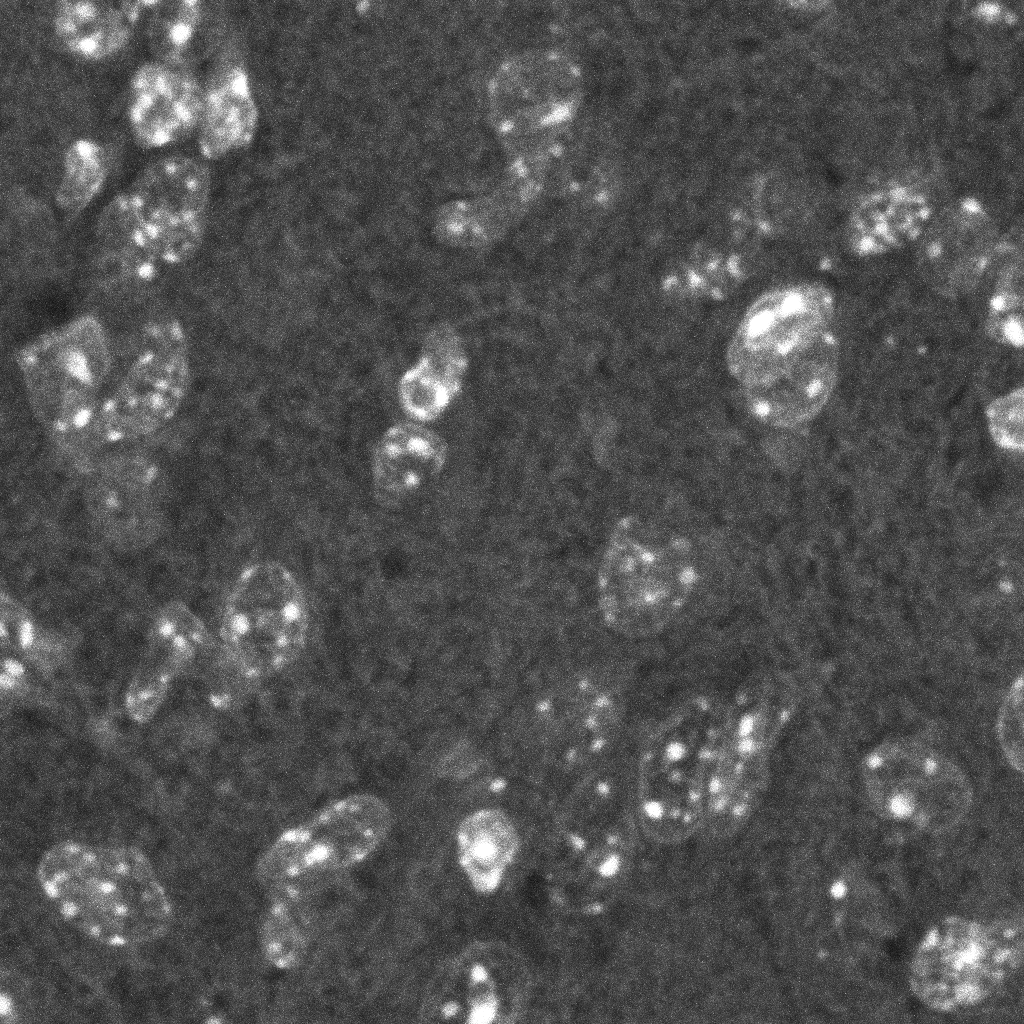

Supplement: Supplementary file 14 — Source data Fig. 1 [file 44318_2024_176_MOESM14_ESM.zip › Figure1/1F/P53_DAPI.tif]

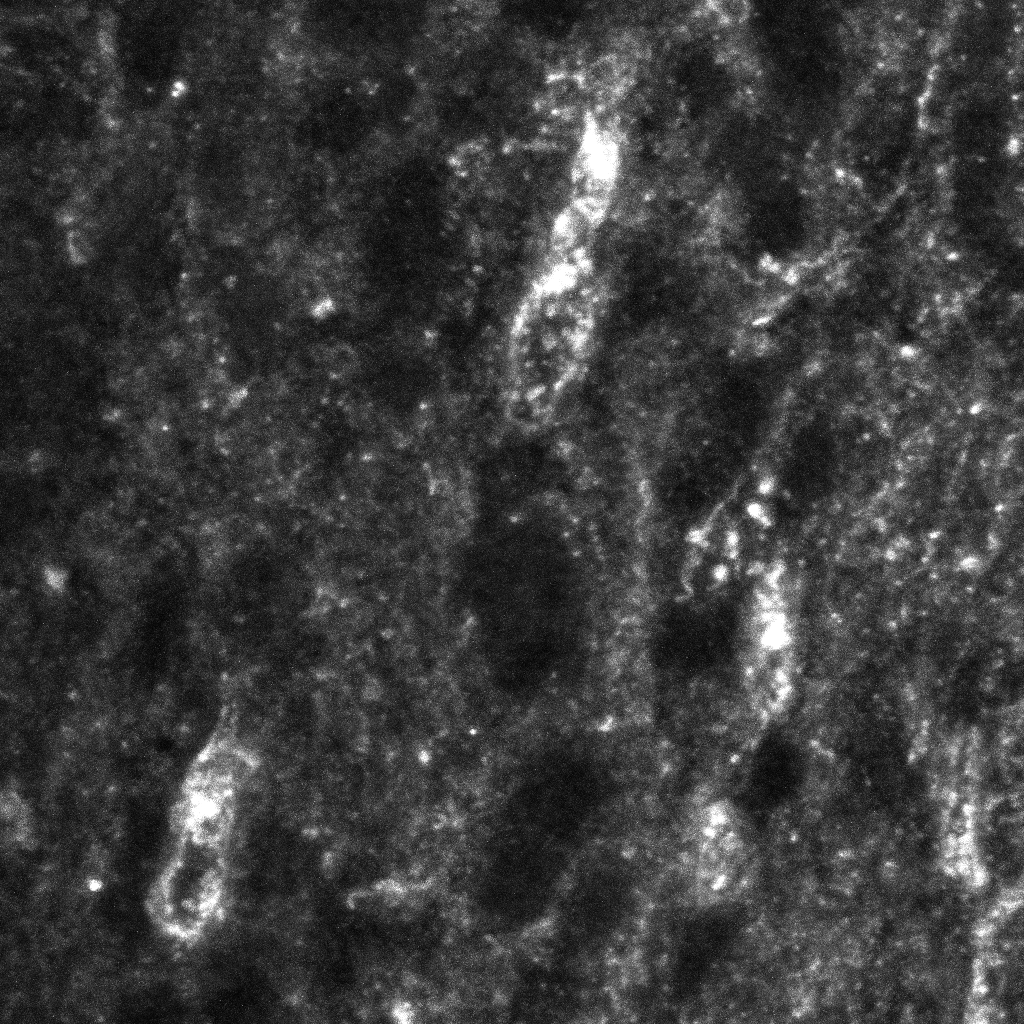

Supplement: Supplementary file 14 — Source data Fig. 1 [file 44318_2024_176_MOESM14_ESM.zip › Figure1/1F/N1IC_CLU.tif]

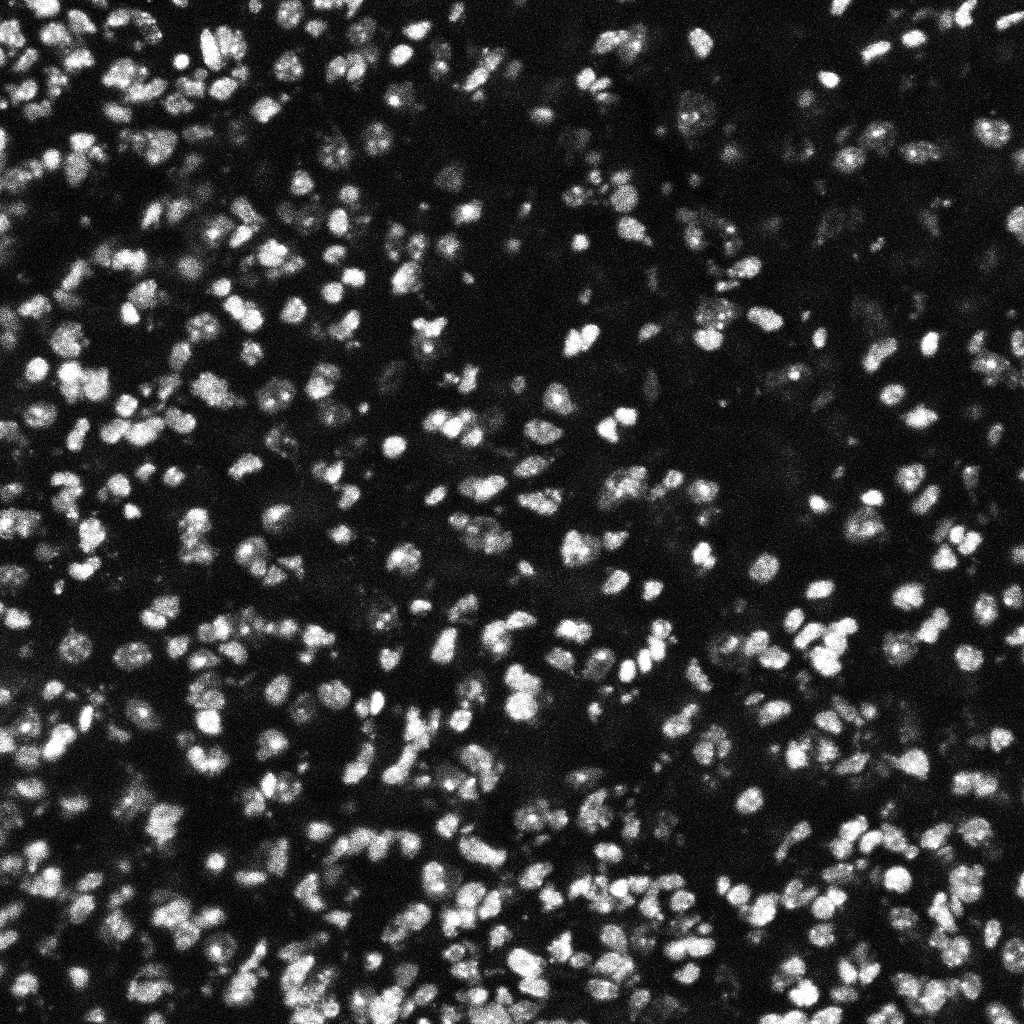

Supplement: Supplementary file 14 — Source data Fig. 1 [file 44318_2024_176_MOESM14_ESM.zip › Figure1/1D/N1IC_DAPI.tif]

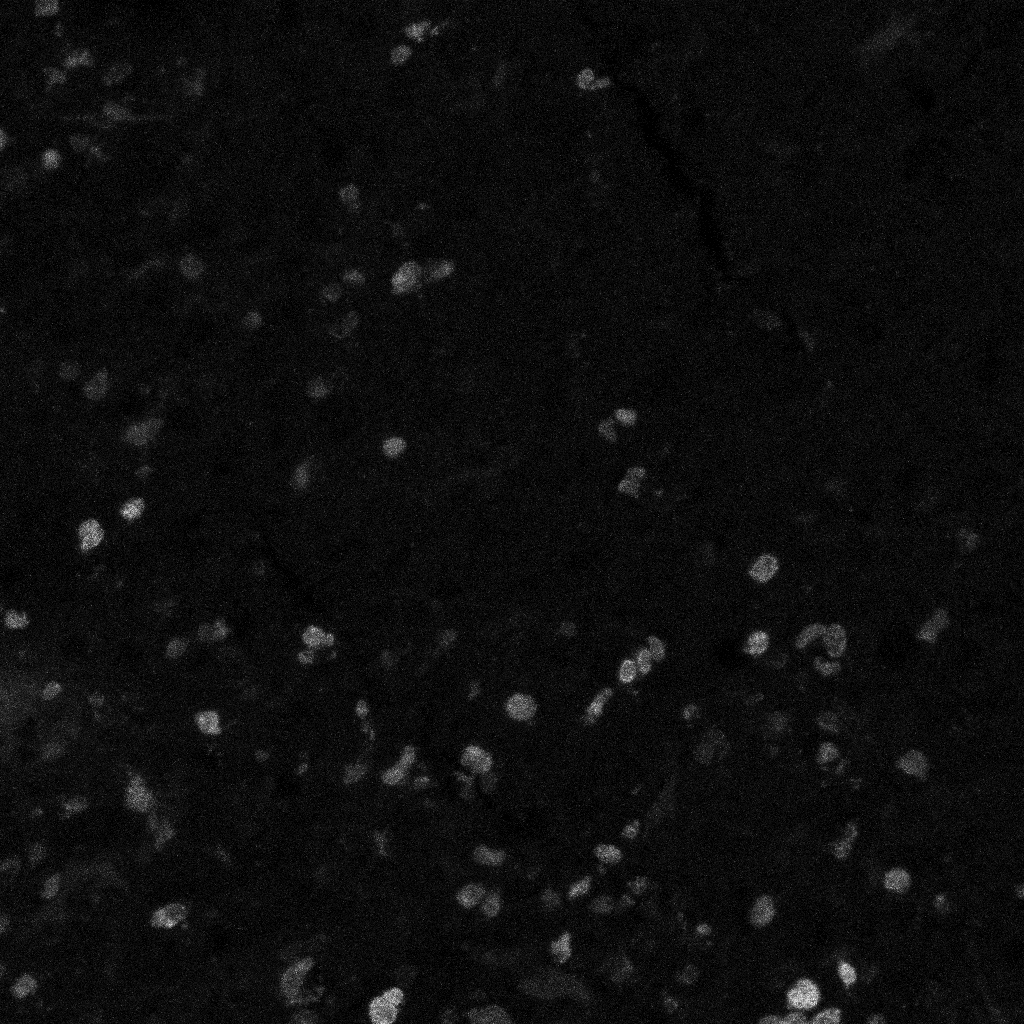

Supplement: Supplementary file 14 — Source data Fig. 1 [file 44318_2024_176_MOESM14_ESM.zip › Figure1/1D/N1IC_Ki67.tif]

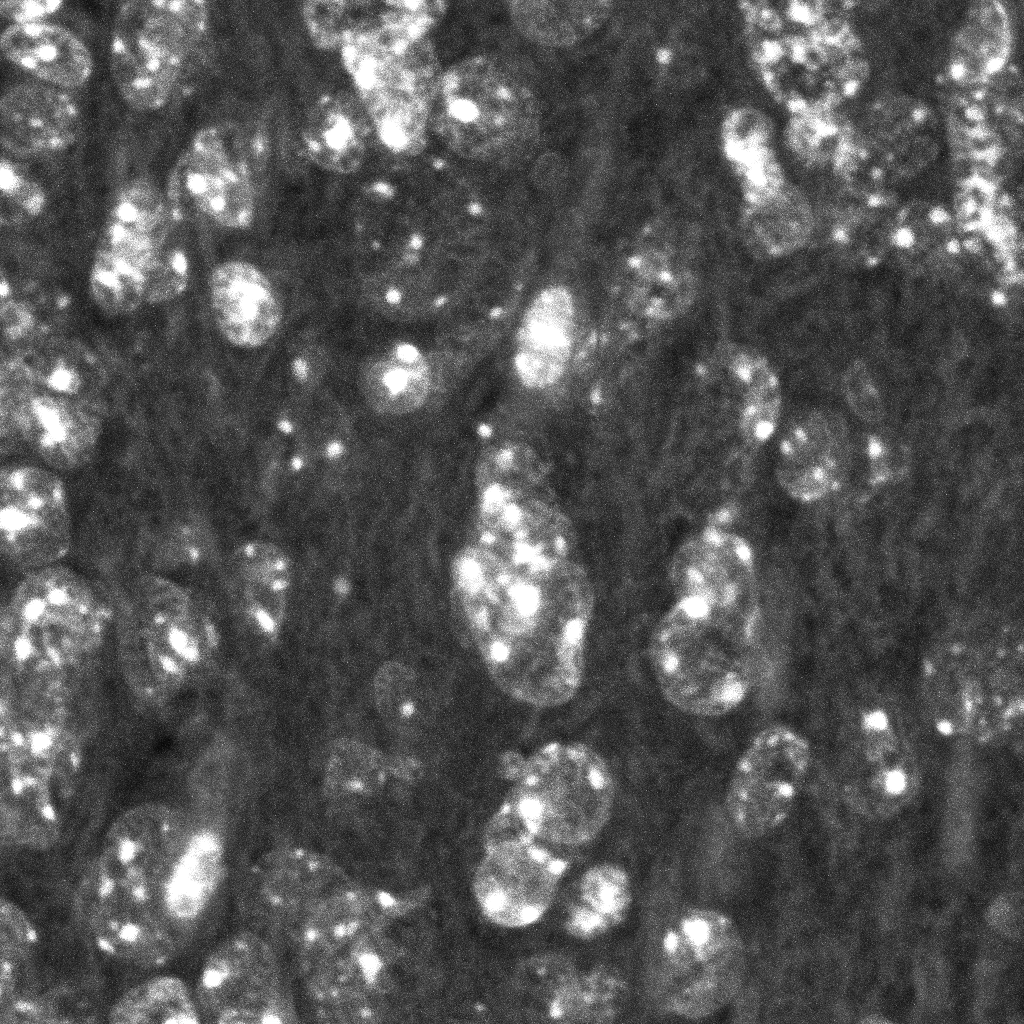

Supplement: Supplementary file 14 — Source data Fig. 1 [file 44318_2024_176_MOESM14_ESM.zip › Figure1/1F/N1IC_DAPI.tif]

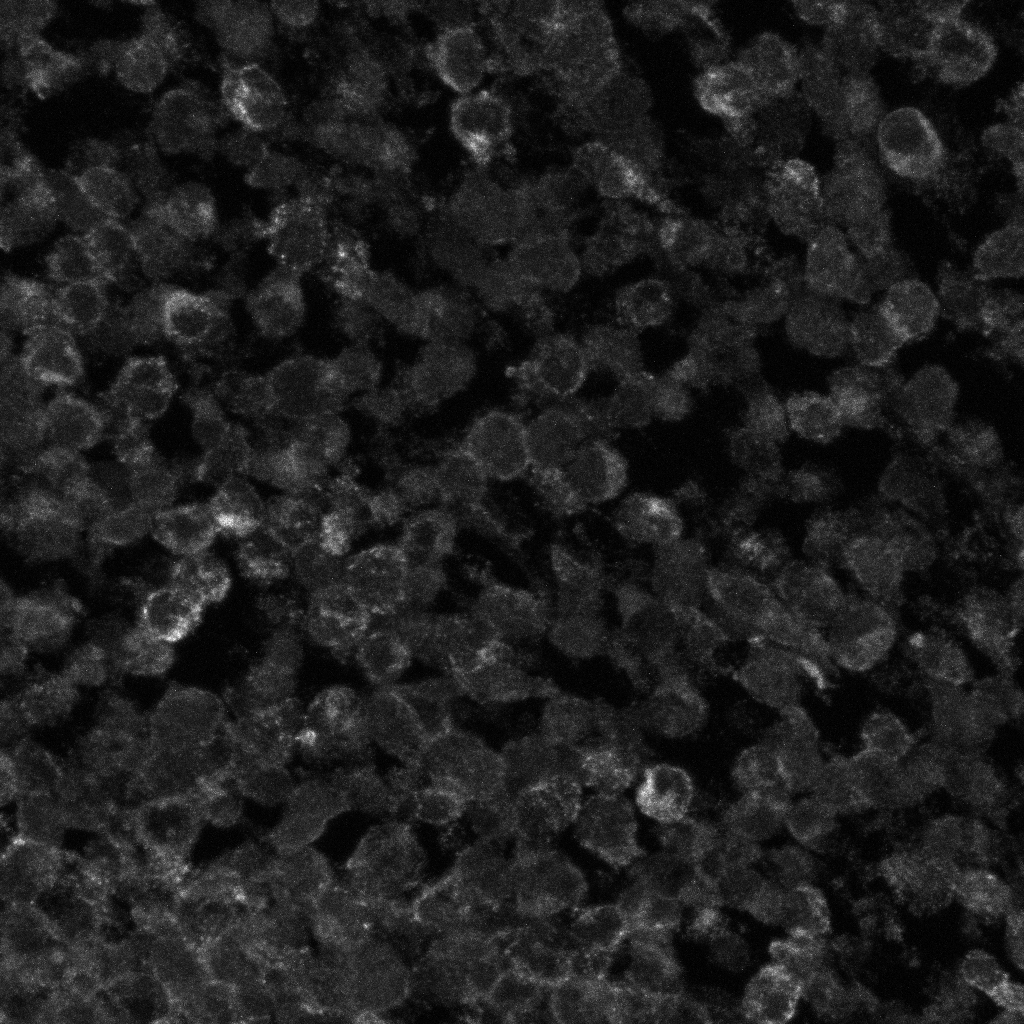

Supplement: Supplementary file 16 — Source data Fig. 4 [file 44318_2024_176_MOESM16_ESM.zip › Figure4/4F/RSL3_TfR.tif]

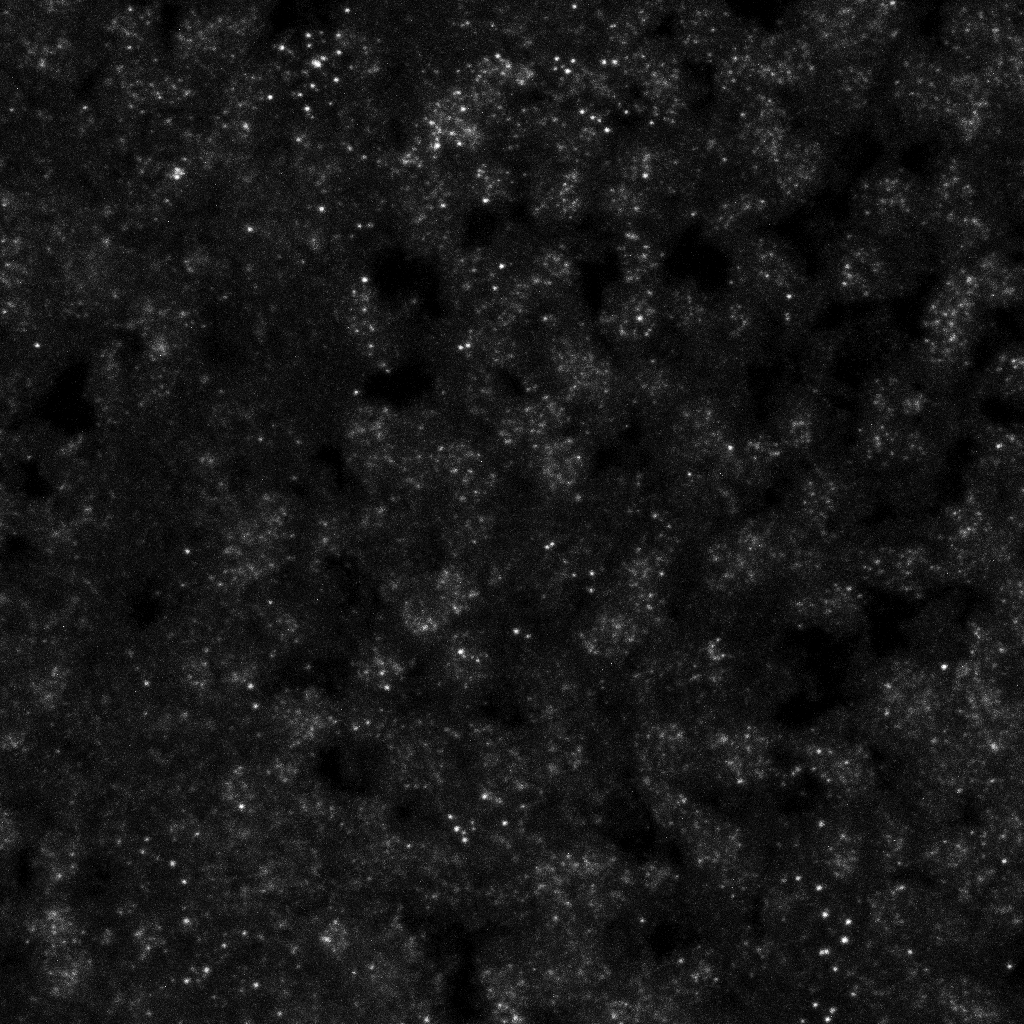

Supplement: Supplementary file 16 — Source data Fig. 4 [file 44318_2024_176_MOESM16_ESM.zip › Figure4/4G/RSL3_CLU.tif]

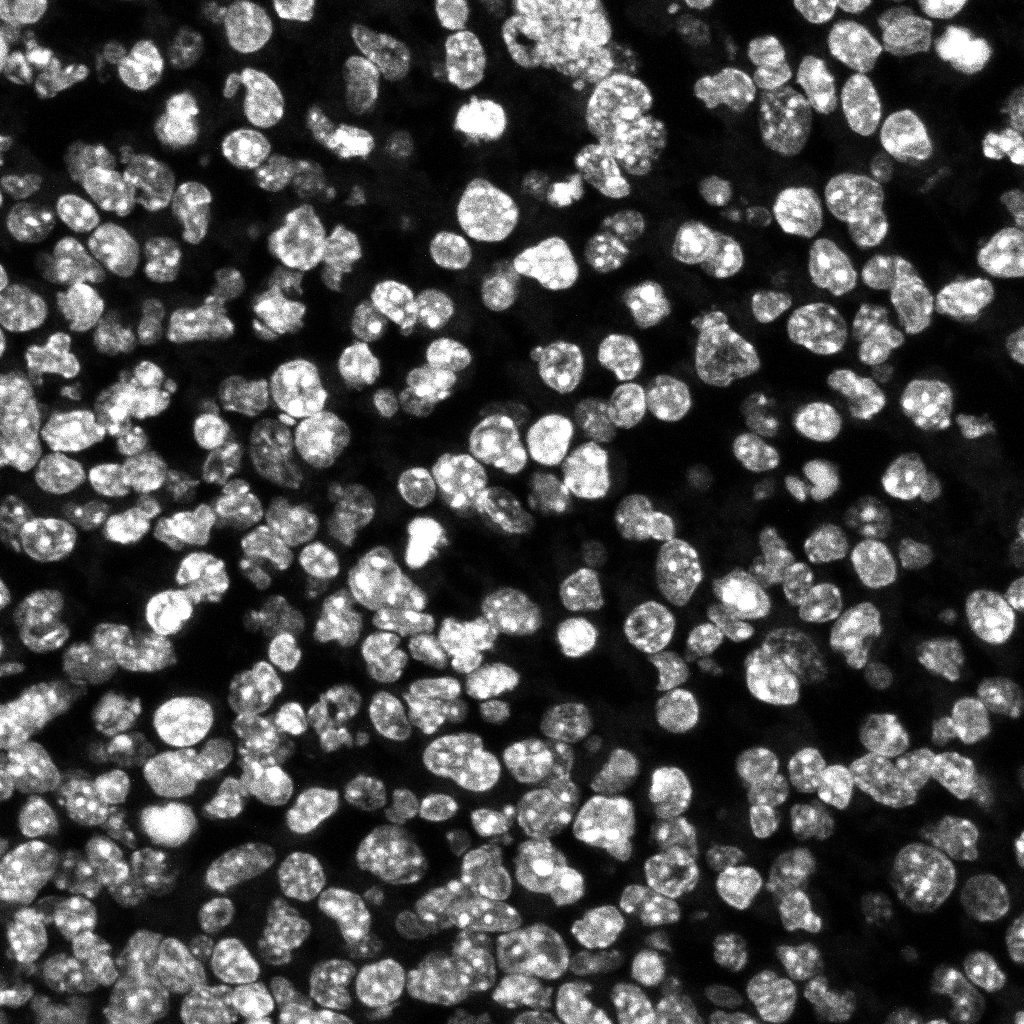

Supplement: Supplementary file 16 — Source data Fig. 4 [file 44318_2024_176_MOESM16_ESM.zip › Figure4/4F/RSL3_DAPI.tif]

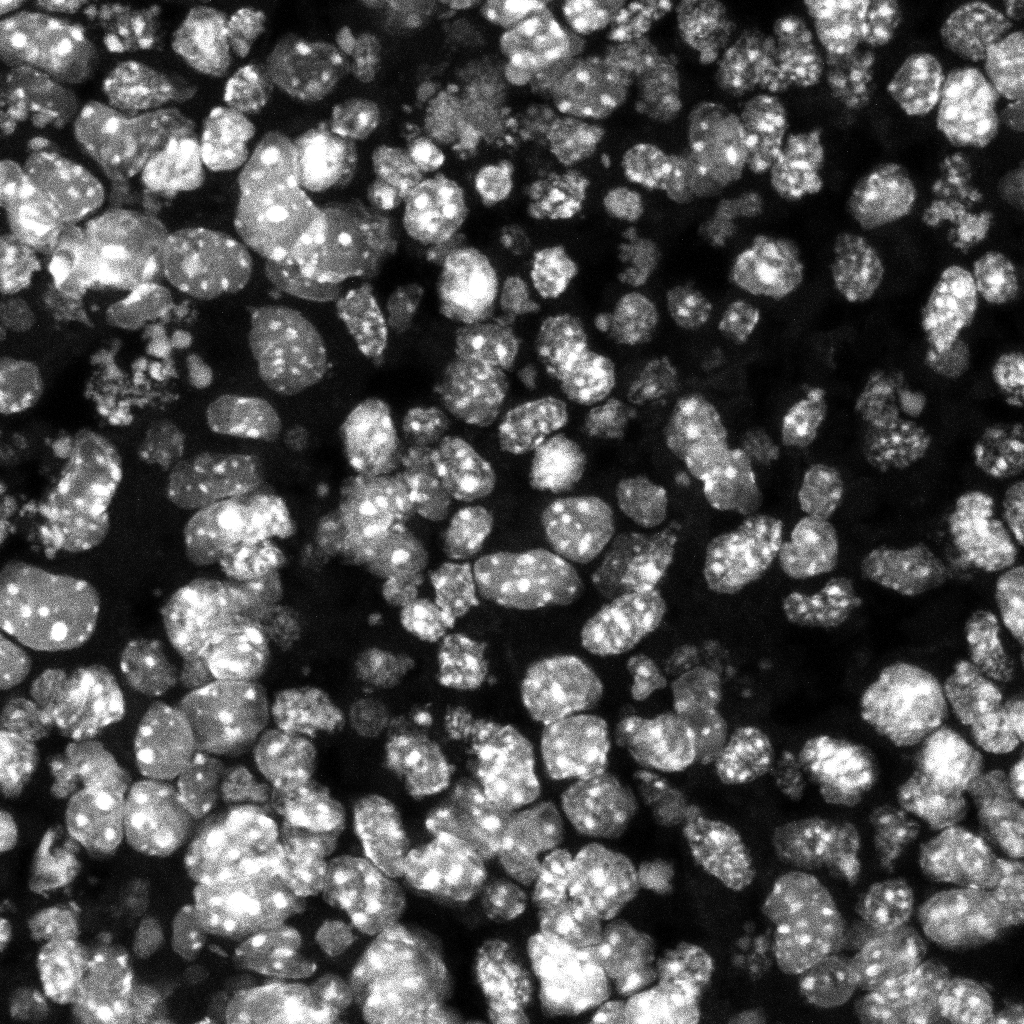

Supplement: Supplementary file 16 — Source data Fig. 4 [file 44318_2024_176_MOESM16_ESM.zip › Figure4/4G/RSL3_DAPI.tif]

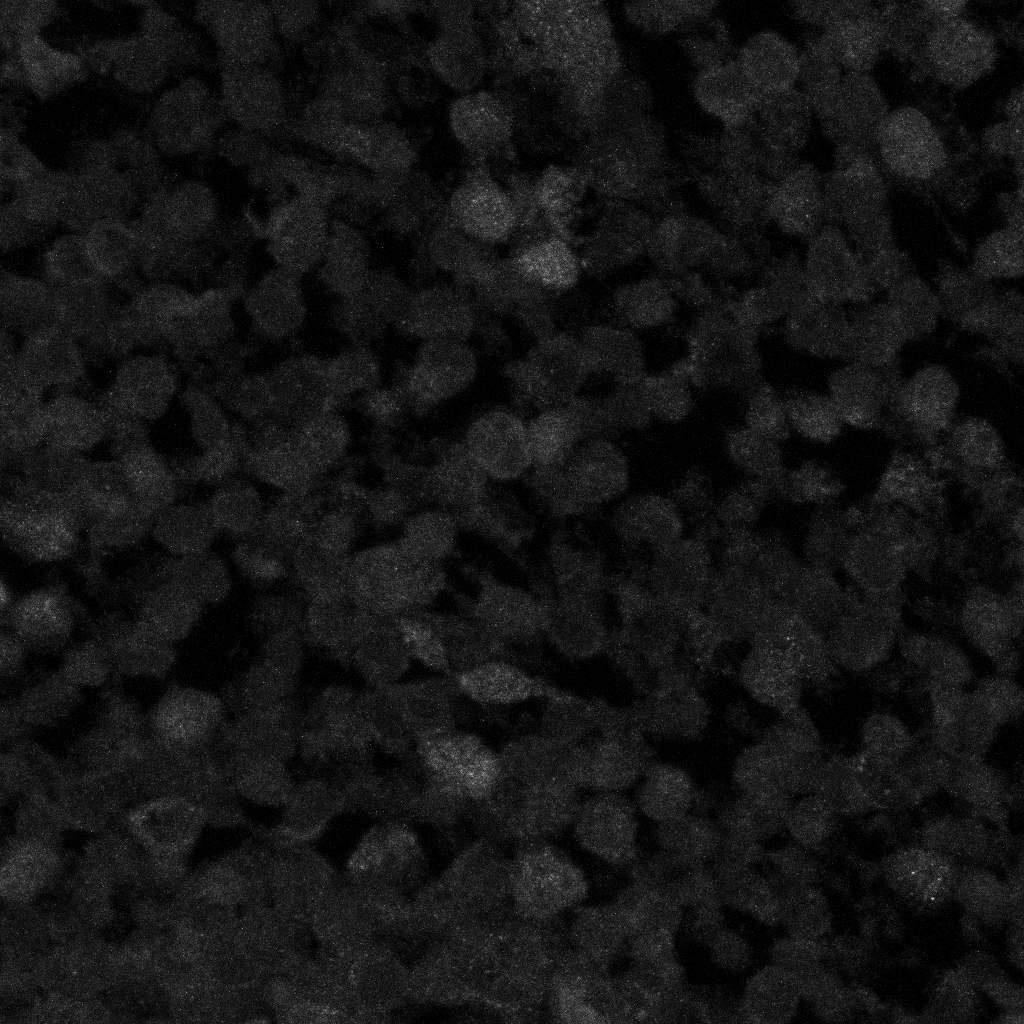

Supplement: Supplementary file 16 — Source data Fig. 4 [file 44318_2024_176_MOESM16_ESM.zip › Figure4/4F/RSL3_Top2a.tif]

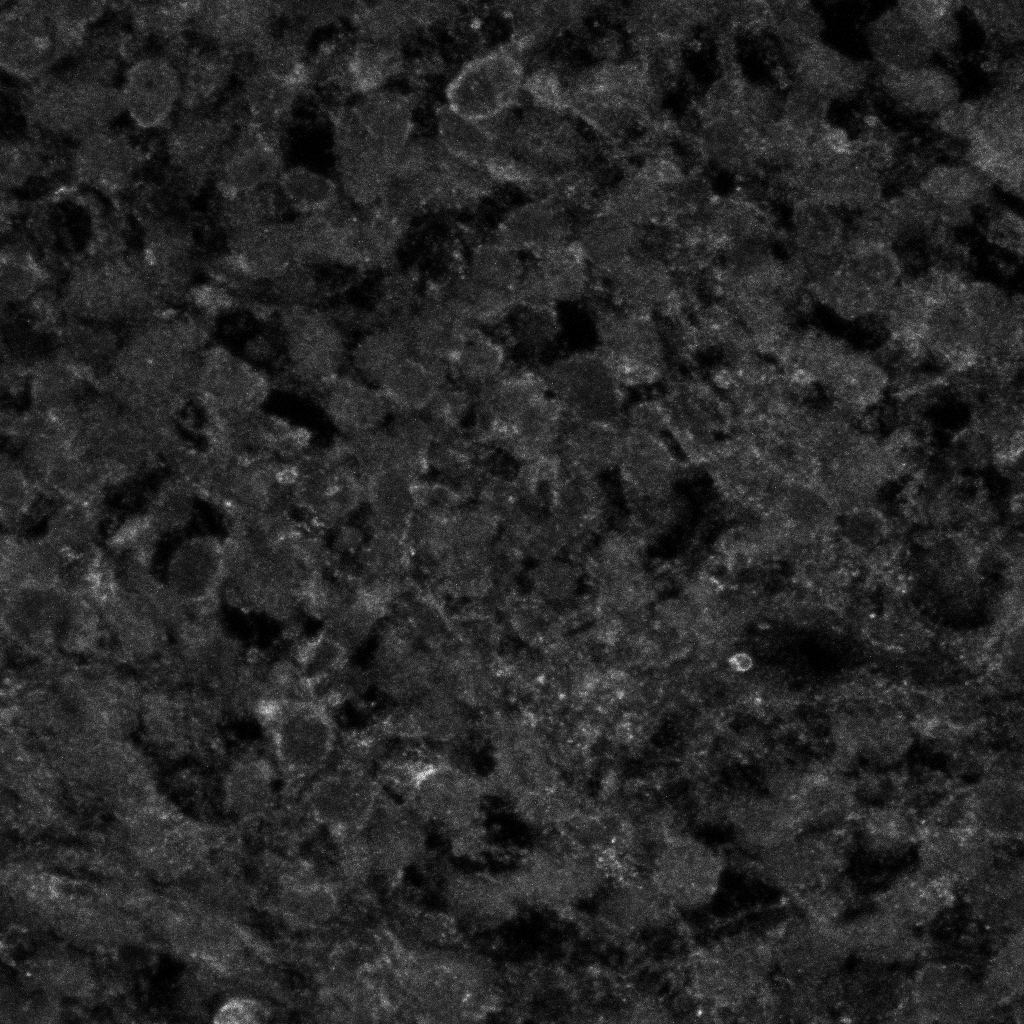

Supplement: Supplementary file 16 — Source data Fig. 4 [file 44318_2024_176_MOESM16_ESM.zip › Figure4/4F/Vehicle_TfR.tif]

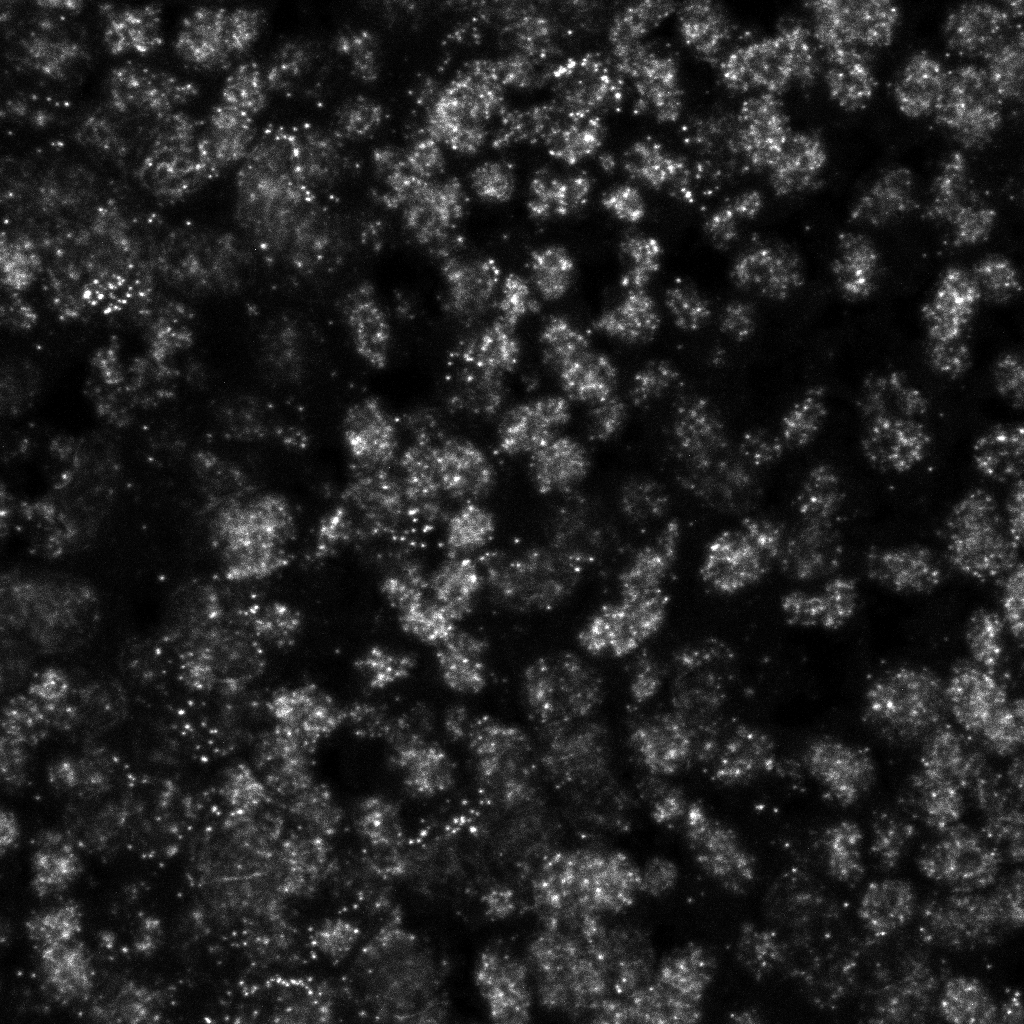

Supplement: Supplementary file 16 — Source data Fig. 4 [file 44318_2024_176_MOESM16_ESM.zip › Figure4/4G/RSL3_ptprz1.tif]

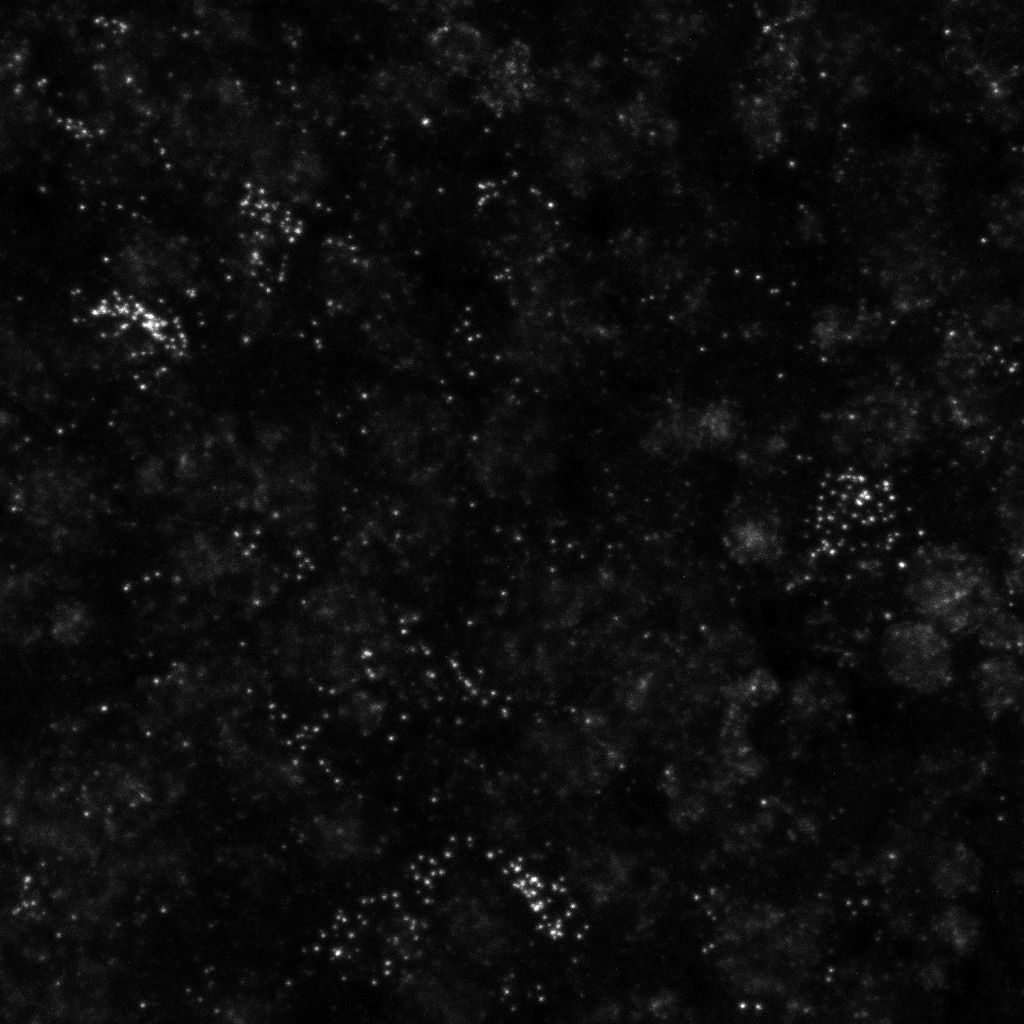

Supplement: Supplementary file 16 — Source data Fig. 4 [file 44318_2024_176_MOESM16_ESM.zip › Figure4/4G/Vehicle_CLU.tif]

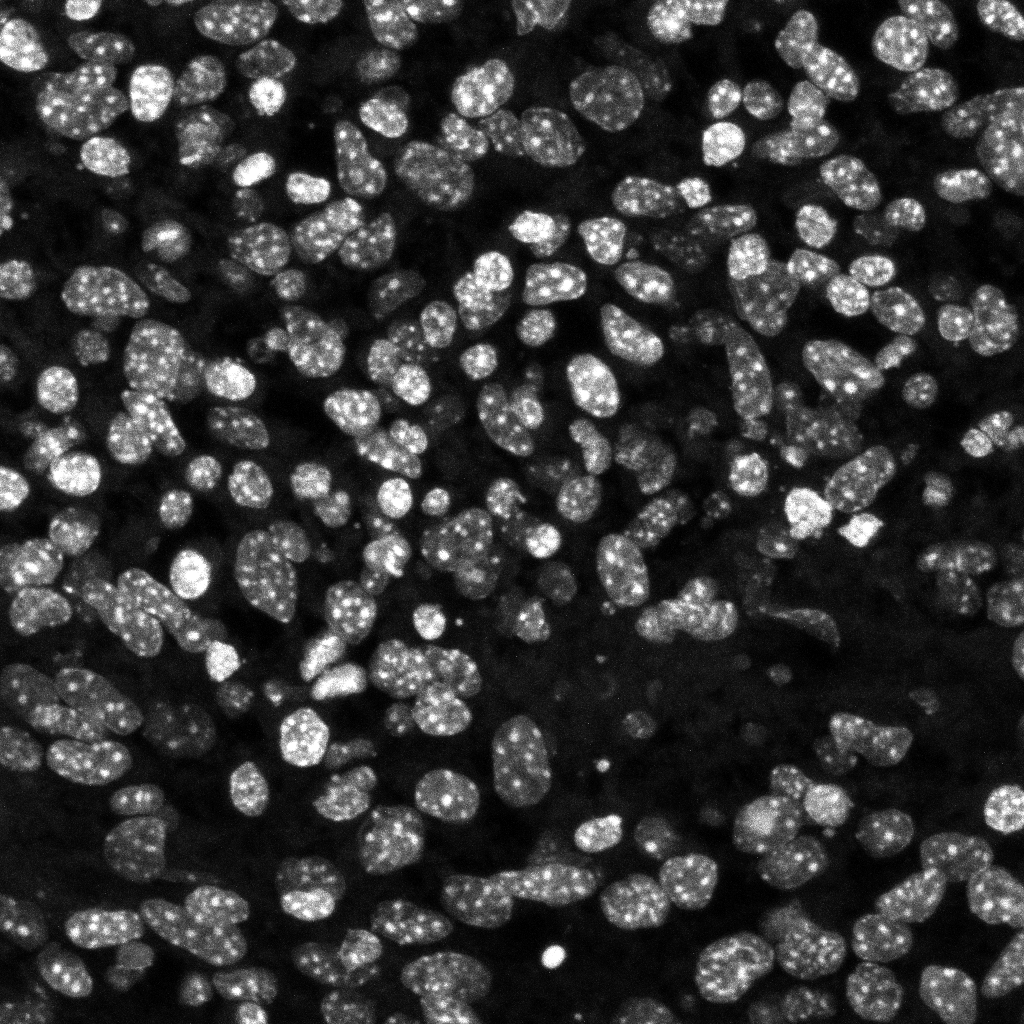

Supplement: Supplementary file 16 — Source data Fig. 4 [file 44318_2024_176_MOESM16_ESM.zip › Figure4/4F/Vehicle_DAPI.tif]

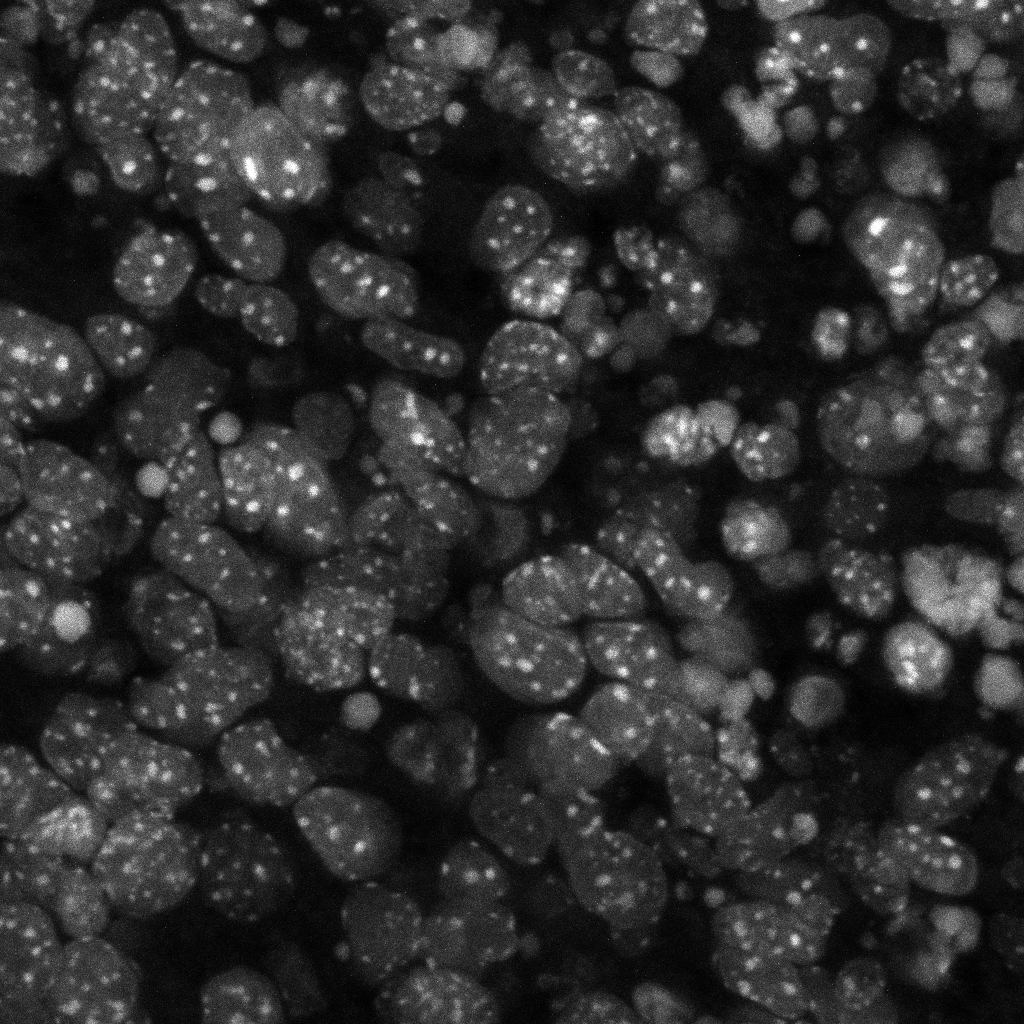

Supplement: Supplementary file 16 — Source data Fig. 4 [file 44318_2024_176_MOESM16_ESM.zip › Figure4/4G/Vehicle_DAPI.tif]

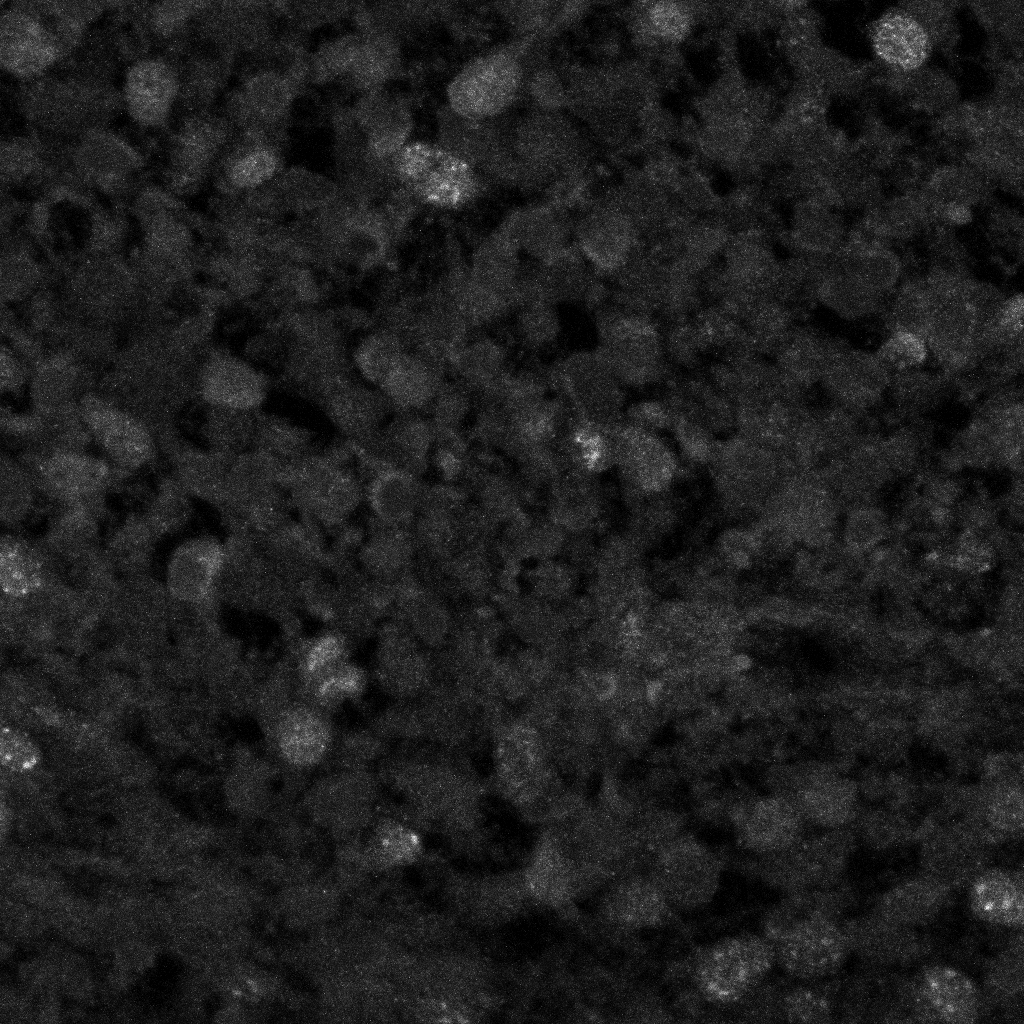

Supplement: Supplementary file 16 — Source data Fig. 4 [file 44318_2024_176_MOESM16_ESM.zip › Figure4/4F/Vehicle_Top2a.tif]

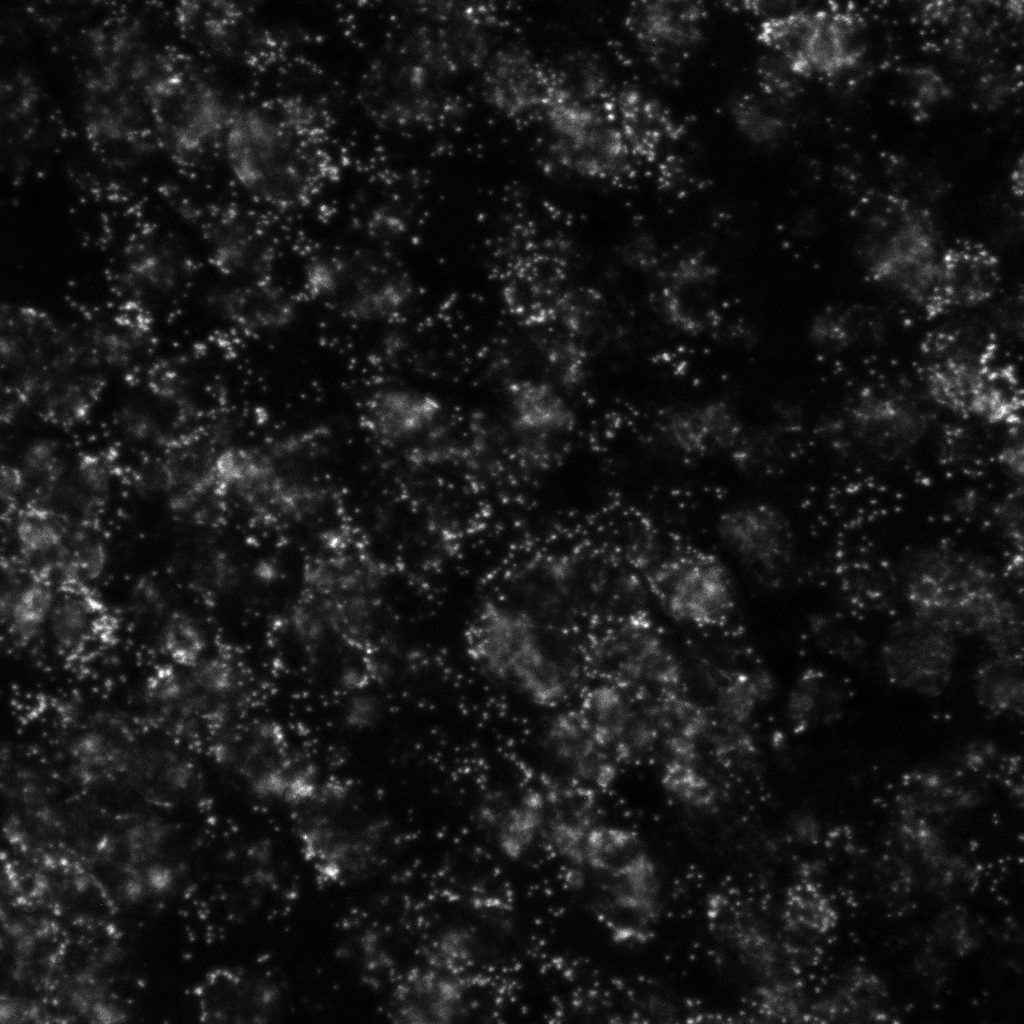

Supplement: Supplementary file 16 — Source data Fig. 4 [file 44318_2024_176_MOESM16_ESM.zip › Figure4/4G/Vehicle_ptprz1.tif]

Figure S5f

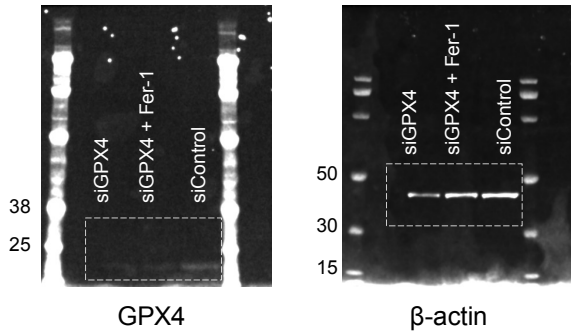

Figure S6d

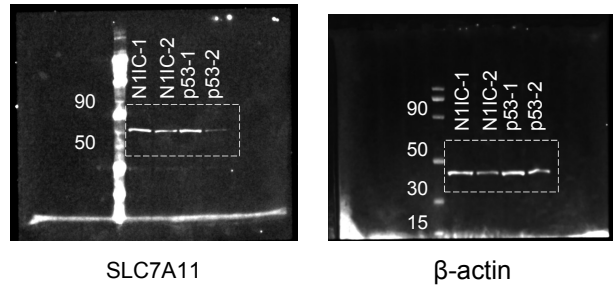

Supplement: Supplementary file 20 — Appendix Figure Source Data [file 44318_2024_176_MOESM20_ESM.pdf]
